# Occupational Exposure and Environmental Release: The Case Study of Pouring TiO_2_ and Filler Materials for Paint Production

**DOI:** 10.3390/ijerph18020418

**Published:** 2021-01-07

**Authors:** Ana Sofia Fonseca, Anna-Kaisa Viitanen, Tomi Kanerva, Arto Säämänen, Olivier Aguerre-Chariol, Sebastien Fable, Adrien Dermigny, Nicolas Karoski, Isaline Fraboulet, Ismo Kalevi Koponen, Camilla Delpivo, Alejandro Vilchez Villalba, Socorro Vázquez-Campos, Alexander Christian Østerskov Jensen, Signe Hjortkjær Nielsen, Nicklas Sahlgren, Per Axel Clausen, Bianca Xuan Nguyen Larsen, Vivi Kofoed-Sørensen, Keld Alstrup Jensen, Joonas Koivisto

**Affiliations:** 1 National Research Centre for the Working Environment (NRCWE), DK-2100 Copenhagen, Denmark; alj@nfa.dk (A.C.Ø.J.); shn@nfa.dk (S.H.N.); nms@nfa.dk (N.S.); PAC@nfa.dk (P.A.C.); bxl@nfa.dk (B.X.N.L.); VKS@nfa.dk (V.K.-S.); KAJ@nfa.dk (K.A.J.); joonas.apm@gmail.com (J.K.); 2 Finnish Institute of Occupational Health, FI-00032 Työterveyslaitos, Finland; anna-kaisa.viitanen@ttl.fi (A.-K.V.); tomi.kanerva@ttl.fi (T.K.); Arto.Saamanen@ttl.fi (A.S.); 3 Caractérisation de l’Environnement (CARA), INERIS, 93310 Verneuil-en-Halatte, France; Olivier.AGUERRE-CHARIOL@ineris.fr (O.A.-C.); Sebastien.FABLE@ineris.fr (S.F.); Adrien.DERMIGNY@ineris.fr (A.D.); Nicolas.KAROSKI@ineris.fr (N.K.); Isaline.FRABOULET@ineris.fr (I.F.); 4 Clean Air Technologies, FORCE Technology, DK-2605 Brøndby, Denmark; ik@force.dk; 5 Human & Environmental Health & Safety, LEITAT Technological Center, 08005 Barcelona, Spain; cdelpivo@leitat.org (C.D.); alex.vilchezvillalba@gmail.com (A.V.V.); svazquez@leitat.org (S.V.-C.); 6 ARCHE Consulting, B-9032 Ghent, Belgium; 7 Air Pollution Management, DK-2100 Copenhagen, Denmark; 8 Institute for Atmospheric and Earth System Research (INAR), University of Helsinki, FI-00014 UHEL Helsinki, Finland

**Keywords:** paint industry, particle emissions, occupational exposure, environmental release, exposure determinants, powder handling

## Abstract

Pulmonary exposure to micro- and nanoscaled particles has been widely linked to adverse health effects and high concentrations of respirable particles are expected to occur within and around many industrial settings. In this study, a field-measurement campaign was performed at an industrial manufacturer, during the production of paints. Spatial and personal measurements were conducted and results were used to estimate the mass flows in the facility and the airborne particle release to the outdoor environment. Airborne particle number concentration (1 × 10^3^–1.0 × 10^4^ cm^−3^), respirable mass (0.06–0.6 mg m^−3^), and PM_10_ (0.3–6.5 mg m^−3^) were measured during pouring activities. In overall; emissions from pouring activities were found to be dominated by coarser particles >300 nm. Even though the raw materials were not identified as nanomaterials by the manufacturers, handling of TiO_2_ and clays resulted in release of nanometric particles to both workplace air and outdoor environment, which was confirmed by TEM analysis of indoor and stack emission samples. During the measurement period, none of the existing exposure limits in force were exceeded. Particle release to the outdoor environment varied from 6 to 20 g ton^−1^ at concentrations between 0.6 and 9.7 mg m^−3^ of total suspended dust depending on the powder. The estimated release of TiO_2_ to outdoors was 0.9 kg per year. Particle release to the environment is not expected to cause any major impact due to atmospheric dilution

## 1. Introduction

Recent studies have shown that the release of ultrafine particles to air (UFPs; defined as the fraction of fine particles with an electrical mobility diameter ≤ 100 nm) originating from handling of conventional non-nano classified materials may be substantial [1,2,3]. They may even dominate the release of UFPs also in industries using manufactured nanomaterials (NMs; in the EU defined as materials in which 50% of the particles by number have one or more external dimensions in the range of 1–100 nm). In most cases, workers are exposed to a heterogeneous mixture of different particles, which can make the quantitative exposure characterization and risk assessment very complex [1,4].

The paint and coating manufacturers are well known as a highly important downstream user of large amounts of NMs and conventional non-nano materials [5,6]. By 2022, the paint industry alone is expected to reach a global value of 209.4 billion U.S. dollars [7]. Paint is considered a suspension of organic and inorganic particulate materials (e.g., cellulose, TiO_2_, ZrO_2_, ZnO, Ag, and CeO_2_) in a liquid composed of a binder (resin), a volatile solvent or water, and additives to impart protective, durability, decorative, dirt repellence, color, gloss coating, or other properties to a substrate [8,9,10]. In general, the paint manufacturing process involves powder handling, pouring, mixing, dispersing, thinning and adjusting, filling of containers, cleaning operations, and warehousing [11]. Vast volumes of the materials added in conventional paints are handled as dry powders, which may fall under the EU NM definition or contain a fraction of nanoparticles and release UFP during their handling [6].

Previous studies have shown that paint and coating manufacturers emitted 6000, 600, and 400 tons of VOCs (volatile organic compounds), PM_10_ (mass of particulate matter collected with a 50% cut-point of 10 µm in aerodynamic diameter, *D_ae_*), and PM_2.5_ (50% cut-point of 2.5 µm *D_ae_*), respectively to the outdoor environment [12]. Van Broekhuizen et al. [5] also confirmed a significant UFP release and an associated worker exposure reaching >1 × 10^5^ cm^−3^ during manufacturing of conventional non-nano waterborne paint, which involved pouring conventional pigment grade TiO_2_ and fillers such as calcium carbonate (CaCO_3_) and talc (Mg_3_Si_4_O_10_(OH)_2_). Koponen et al. [13] found worker exposures to PM_1_ (50% cut-point of 1 µm *D_ae_*) in different paint producing facilities varying from 0.2 to 0.8 mg m^−3^. Hence, there is evidence that paint production can result in a significant worker exposure and environmental release of UFP and fine pigment and filler dust particles.

There is also evidence that many of the pigments and fillers used in paints can cause diverse negative health effects after lung exposure, e.g., [14,15,16,17,18]. The risk of pulmonary hazard appears to be generally higher in connection with exposure to NM and UFP as compared with coarser particles and similarities can be found between the effects of NM and UFP in the ambient air pollution [19]. The European Agency for Safety and Health at Work [20] consider UFP as one of the major risks in workplace microenvironments. Considering public health, exposure to the general ambient and indoor air pollution has also been linked to adverse health effects including cancer, respiratory, cardiovascular, and nervous system diseases [21,22,23,24,25]. PM_2.5_ air pollution has been ranked as the 6th highest risk factor for early death [26]. While fossil fuel combustion particles are considered to play the most important role in the observed public health effect of ambient air pollution, industrial sources can have important local influence, e.g., [27,28]. Hence, it is important to understand the potential pollution impact of an industrial plant on its near-field surroundings. This, both in regards to potential direct environmental exposure and accumulation of persistent pollutants, which can affect both humans and ecosystems [29,30].

Occupational and environmental exposure assessments are therefore important steps in a risk management program in order to maintain the workplace exposure below limit values and assure the protection of the environment [31,32]. High quality industrial exposure studies are also needed to further understand the exposure determinants, which are critical for occupational and environmental exposure model development and testing [33,34,35,36,37,38,39].

In this study, a workplace field measurement campaign was performed at a paint factory producing three different paint batches (12.4–14.5 tonnes per batch). Work tasks, such as handling solvents and pouring of conventional pigments and fillers, with potential impacts on human health and environment, were studied in different pouring lines. The main objective was to demonstrate a holistic approach in safety assessment by evaluating: (i) workers personal exposures to PM in terms of total number, mass, and lung deposited surface area concentrations; (ii) mass flows of the airborne particle emissions in the near-field/far-field (NF/FF) and via exhaust air ducts; and (iii) environmental exposure.

## 2. Materials and Methods

### 2.1. Work Environment and Measurement Locations

**Figure 1:**
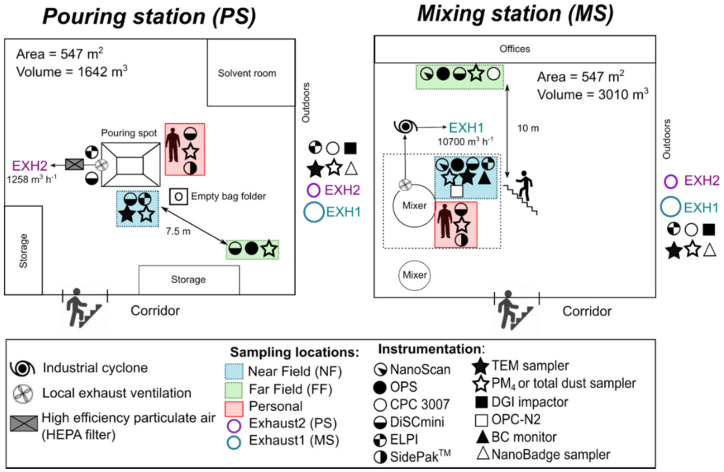
Layout of the working environment and location of instrumentation and samples.

Particle and gas measurements were conducted at a paint manufacturer located in the vicinity of Copenhagen (Denmark) from 29 January to 2 February 2018 during the production of three different paint batches. In two different pouring lines, named hereafter as mixing station (MS) and pouring station (PS), eight different powders (pigments and fillers) were manually poured into the hopper by one worker. In addition to the use of local exhaust ventilation systems at workstations, the facility was naturally ventilated where the outdoor replacement air entered across the building shell and through open doors. The layout of the working environment and placement of the measurement devices and samplers are shown in Figure 1 and photos of the stations, stack emission, and environmental measurements are shown in Supplementary Figure S1.

Particles in the breathing zone (BZ) were measured with the personal measurement instruments attached in a carry bag to the worker’s shoulder. At the stationary NF measurement points in PS and MS the inlets of the instruments and the samplers were approximately at a height of 1.5 m and 0.5 m from the powder pouring activities. Therefore, the NF was defined as the volume around the process activity where the worker stands during the work process. At the stationary FF measurement points, the instruments and samplers were placed from 7.5 to 10 m from the NF locations (Figure 1).

In the MS, small bags (25 kg) with TiO_2_ pigment (Tioxide TR81, Huntsman P&A UK Ltd, Hartlepool, UK), modified alumino-silicate (OpTiMat^®^ 2550, Imerys, Barcelona, Spain), and microspheres (Expancel, Akzo Nobel, Bohus, Sweden) were opened with a knife and manually emptied by pouring directly into a mixing tank from the edge of an opening area of 0.3 m^2^ and a resulting drop height of 1.4 m inside the tank (Supplementary Figure S1a). This mixing tank had local exhaust ventilation (LEV) located under the pouring point attached to the funnel leading to the mixer. This LEV was connected to the mixing stack (EXH1; Figure 1), which had an exhaust air velocity of 5 m s^−1^ and a volumetric dry flow rate of 10,700 m^3^ h^−1^ (Supplementary Figure S1b).

In the PS, powders were poured either from small bags (SBs; 25 kg) or big bags (BBs; 500 kg) through a quadratic opening with an area of 1.27 m^2^ (Supplementary Figure S1c) and conducted via tubes into a mixing tank (Supplementary Figure S1a). The SBs were opened with a knife and manually poured from the edge of the tank and an internal tank drop height of 1 m whereas BBs were lifted with an electric forklift above the pouring funnel. Empty bags were introduced into a disposal container (Supplementary Figure S1c). The PS had local exhaust ventilation (LEV) at the rim along three sides of the pouring inlet. The exhaust duct of this LEV (duct diameter = 125 mm; mean air velocity = 13 m s^−1^; and volumetric flow rate = 576 m^3^ h^−1^) was connected to the pouring stack (EXH2; Figure 1) with a registered exhaust air velocity of 5 m s^−1^ and a volumetric dry flow rate of 1258 m^3^ h^−1^ (Supplementary Figure S1b). Pouring activities in the PS involved the handling of the followed five materials (Table 1): (i) calcined clay (PoleStar™ 200P, Imerys, Cornwall, UK); (ii) calcined kaolinite (Ultrex 96, BASF, Ludwigshafen, Germany); (iii) Talc (Finntalc M15, Mondo Minerals B.V., Helsinki, Finland ); (iv) dolomite (Microdol 1, Norwegian Talc AS, Fjell, Norway); and (v) calcite CaCO_3_ (Socal P2, Solvay Chemicals International SA, Brussels, Belgium).

### 2.2. Raw Materials Characterization

The following characterization was made for the pigment and filler powders:

- Primary particle size, morphology, and elemental composition by scanning electron microscopy (SEM; using a FEI Quanta 200 microscope), operating at an accelerating voltage of 1–2 kV and at magnifications between 20,000× and 50,000×, and transmission electron microscopy (TEM; using a Jeol JEM 1400 Plus microscope), operating at an accelerating voltage of 120 kV, and coupled to an energy dispersive X-ray spectroscopy (EDS; AZTEC from Oxford Instruments). The powders were dispersed in aqueous media with a small amount of ethanol for deagglomeration and deposited on a Ni TEM grids;
- Specific surface area (SSA) analysis by using the Brunauer–Emmett–Teller (BET) method with nitrogen absorption using an Autosorb-1-MP (Quantachrome, Boynton Beach, FL, USA; [43]);
- Dustiness in terms of respirable mass fraction, by using the small rotating drum (SRD; EN17199-4:2019 [41]), respirable dust sampling using GK2.69 cyclone, and size distribution analysis using an electrical low-pressure impactor (ELPI; Dekati model ELPI and ELPI+, Dekati Ltd., Kangasala, Finland);
- Bulk density according to EN17199-1:2019 [44] using a measuring cylinder of known volume of 10 cm^3^ and an analytical balance with a resolution of 0.01 g.

The physicochemical characteristics, and current occupational exposure limits (OEL) are described in Table 1 for each material. According to the manufacturer, the particle sizes *d_50_* varies from 250 for TiO_2_ to >5 µm for talc, dolomite, and clays. The main organic solvents and additives added to the mixer before and after the powders were white spirit, high boiling esters (coalescent agents), glycols, and glycol ethers. Other materials such as different types of cellulose were also added to these paint batches. However, these events were not monitored due to the small amounts used.

### 2.3. Measurement Strategy

The measurement strategy adopted in this study followed the Tier 3 approach for particle exposure assessment published by the Organisation for Economic Co-operation and Development [45] and EN 17058:2018 [46]. It included real-time particle monitoring combined with collection of samples for gravimetric, morphological, and chemical analysis during working and non-working periods.

For background (BG) discrimination (particles from sources other than the target process), a combined approach of temporal and spatial analysis was adopted [32]. The non-working periods were used to define the BG concentrations at all measurement points by using the measurements obtained prior the target activity in the paint manufacturing facility.

### 2.4. Particle Monitoring and Sampling Techniques

The measurements included real-time monitoring of particle concentrations and size distributions, collection of particles on filter samplers, and TEM samples collected simultaneously from NF, FF, BZ, and stacks (Figure 1). The following real-time particle monitors were used:

- Particle mobility size distributions were measured by NanoScan (NS; TSI NanoScan model 3091, TSI Inc., Shoreview, MN, USA) for particles from 10 to 420 nm in 60 s intervals [47,48].
- Optical particle sizer (OPS; TSI model 3330, TSI Inc., Shoreview, MN, USA) was used to measure the optical particle size distributions in 16 channels from 0.3 to 10 μm in 60 s intervals [49,50,51].
- Aerodynamic particle size distributions were measured using an electrical low-pressure impactor (ELPI; Dekati model ELPI and ELPI+, Dekati Ltd., Kangasala, Finland) in 14 size channels between 6 nm or 7 nm and 10 µm with 1 s intervals [52].
- Portable condensation particle counter (CPC; TSI model 3007, TSI Inc., Shoreview, MN, USA) were used to measure the total particle number concentration from 10 nm to >1 μm in 1 s time resolution [53,54].
- Miniature diffusion size classifiers (DiSCmini (DM); Testo SE and Co. KGaA, Lenzkirch, Germany) were used to measure total particle number, mean particle diameter, and the lung deposited surface area (LDSA) of particles with modal diameter in the range of 10–300 nm with 1 s time resolution [55]. To avoid artifacts due to coarse particles, the DM was equipped with an inlet separator with a cutoff diameter of 700 nm.
- Aerosol black carbon detector (BC; Berkeley; [56]) and aethalometer AE33 (Magee Scientific, Berkeley, CA, USA) were used to measure BC mass concentration with 60 s intervals.
- Alphasense optical particle counter OPC-N2 was used to measure PM_1_, PM_2.5_, and PM_10_, and particle size distributions in 60 s intervals [57,58].
- SidePak^TM^ (model AM520, TSI Inc., Shoreview, MN, USA) were used to measure particles in the size range 0.1–10 µm [59,60].

For the personal DM and SidePak, the particles were sampled through transparent conductive Tygon™ tubing (Saint Gobain Performance Plastics, Courbevoie, France) [61], while electrically conductive silicone sampling lines were used for the rest of the instruments. Diffusional losses for the NS and ELPI sampling lines were corrected according to Cheng (2001) [62].

In the case of total particle number concentrations and particle size distributions, we primarily present the results obtained with the ELPI, because it is the only instrument for which measurements were made at the NF of both MS and PS. However, when ELPI data was not available at the MS, particle emissions were characterized by NanoScan and OPS. The mobility and optical particle number size distributions measured by the NS and OPS were combined to form a wide size-range d*N*/dLog (*D_p_*) particle number size distribution for both NF and FF measurements according to Mølgaard et al. [63]. To make this combination it was assumed that a particle mobility and optical diameter were equivalent even though optical diameter may differ from mobility diameter depending on the particle shape, refractive index, and size [64]. The combined particle size distributions were based on the mobility size concentrations by NS from 10 to 300 nm (15th channel of NS was removed) and optical size concentrations by OPS from 300 nm to 10 µm.

The offline methods utilized in this study comprised:

- Collection of respirable dust (*d_50_* cut size of 4 µm) for gravimetric and inorganic chemical analysis by using Fluoropore™ (Millipore, Billerica, MA, USA) membrane filters 37-mm polytetrafluorethylene (PTFE) with a 0.8-μm pore size mounted in cyclones GK2.69 (BGI Inc., Waltham, MA, USA) or SCC1.062 (BGI Inc., Waltham, MA, USA), connected to portable sampling pumps (Apex2, Casella Inc., Bedford, UK) operating at 4.2 L min^−1^ or 1.05 L min^−1^, respectively [65].
- Note: Particle mass concentrations were gravimetrically determined by pre- and post-weighing the filters collected using an electronic microbalance (Mettler Toledo Model XP6) with ±1 μg sensitivity located in a climate-controlled weighing room (relative humidity (RH) = 50% T = 22 °C). Three blind filters were stored to be used as laboratory blanks to correct for handling and environmental factors.
- Collection of size-fractioned fine dust for gravimetric analysis by using a 4-stage cascade impactor, without pre separator mounted with 47-mm aluminum foils (Dekati^®^ Gravimetric Impactor-DGI, model DGI-1571, Dekati Ltd., Kangasala, Finland) at a flow rate of 70 L min^−1^, which results in calculated *d_50_* cut-off diameters of >2.5, 1.0–2.5, 0.5–1, and 0.2–0.5 μm. An after-filter collected particles <0.2 µm. Weighing was conducted as mentioned above.
- Isokinetic sampling on the quartz filter according to EN 13284-1 (reference method for characterization of TSP) for determination of TSP mass concentrations and combined with adsorption solutions according to EN 14385:2004 [66] (reference method for characterization of heavy metals in atmospheric emission of stationary sources). This filter is then analyzed together with the adsorption solutions in order to determine the concentration of 11 metals (As, Cd, Cr, Co, Cu, Mn, Ni, Pb, Sb, Ti, and V) by means of inductively coupled plasma—optical emission spectrometry (ICP-OES).
- Collection of airborne particles on 400-mesh Cu grids precoated with holey carbon film by using a mini-particle sampler (MPS; Ecomesure; [67]) connected to a pump (Apex2, Casella Inc., Bedford, UK) operating at 0.3 L min^−1^ during 2–5 min sampling time. Aerosol samples collected by MPS were analyzed by TEM (Jeol JEM 1400 Plus microscope), operating at an accelerating voltage of 120 kV, and coupled to an EDS system (AZTEC from Oxford Instruments, High Wycombe, UK). In situ EDS chemical analysis of agglomerates and individual particles were performed with an acquisition time of 100 s.
- Airborne gas-phase organic compounds ((S)VOC) were sampled on Tenax TA with GilAir5 pumps (Gilian, St. Petersburg, FL, USA) for 79–81 min with a flow of 93–110 mL min^−1^ resulting in sampled volumes of 7.9–9.6 L. The Tenax TA tubes were cleaned before sampling in a stream of pure nitrogen at 300 °C for 180 min and 340 °C for 30 min using a sample tube conditioning apparatus (TC-20, Markes International, Llantrisant, UK). The Tenax TA tubes were analyzed by thermal desorption gas chromatography and mass spectrometry (TD-GC–MS) using a Perkin Elmer Turbo Matrix 350 thermal desorber coupled to a Bruker SCION TQ GC-MS system (Bruker Daltonics, Bremen, Germany). Desorption was carried out in a He flow of 1 mL/min at 275 °C for 20 min and desorbed (S)VOCs collected in a cold trap at −20 °C, followed by flash desorption of the cold trap at 275 °C for 1.5 min transferring the (S)VOCs to the GC column. The column was a 5% phenyl polydimethylsiloxane of 30 m × 0.25 mm with 0.25 µm film thickness (VF-5MS, Agilent Technologies, CA, USA). The GC oven program was 40 °C for 2 min, then 20 °C/min to 150 °C hold for 10 min, then 5 °C/min to 275 °C hold for 6 min, and finally 3 °C/min to 300 °C hold for 1 min. The transfer line and the source were kept at 280 °C. The MS was operated with electron ionization (EI) in scan mode (mass range m/z 40–500). Tentative identification of the organic compounds was performed by MS Data Review, Version 8.0.1 (Bruker, Billerica, MA, USA), and NIST/EPA/NIH Mass Spectral Library Version 2.0g, 19 May 2011 (NIST, Gaithersburg, MD, USA). The terpenes were identified using authentic standards as well. The air concentrations of (S)VOCs were semi-quantified using n-decane as a calibration standard.
- Velocity measurements of local exhaust ventilation stacks were measured by a pitot tube.

Particularly for the environmental monitoring and sampling, an original methodology not yet published was followed. Description can be found in the Supplementary Information.

### 2.5. Data Processing

All online instruments were time-synchronized and intercompared overnight, the day before the actual measurements. Worker area exposures in terms of total particle number concentration (*N*) were considered statistically significant when the following approach, described by Asbach et al. and Kaminski et al. [68,69], was fulfilled:

(1) $$WA>\mathrm{BG}+3\cdot \sigma_{\mathrm{BG}}$$

where *WA* is the mean particle number concentrations in the workplace (either NF or BZ) during the pouring activity, BG is the mean spatial background registered concentrations simultaneously at FF, and σ_BG_ is the standard deviation of the BG concentration. However, this approach should be carefully used and interpreted in the presence of secondary sources of particles and especially in small workplaces with poor ventilation systems. In the present study, the combined approach of temporal and spatial analysis allowed us to distinguish the target particles from the background.

The cumulative worker exposure was calculated as an 8-h time weighted average (8 h-TWA). In this study, we calculated the 8 h-TWA based on the workers exposure duration during pouring activities (total daily duration varying from 2.2 to 5.8 h) and the spatial background concentrations (measured in FF) for the remaining hours.

## 3. Results

### 3.1. Raw Materials Characterization

Table 1 summarizes the characteristics of the eight different pigment and filler materials studied in this exposure study. Representative electron micrographs of the eight materials are shown in Supplementary Figure S2 and used for validation or modification of particle characteristics. The characterization data demonstrate a considerable range in BET-SSA values (e.g., dolomite and TiO_2_ with 3.2 and 13 m^2^ g^−1^, respectively), particle grain sizes (0.25 to 30 µm), bulk densities (0.16–1.09 g cm^−3^), and particle shapes (e.g., plate talc, plate clays, rod calcite, and spherical TiO_2_ and dolomite). Therefore, establishing a general relationship between emission patterns and materials physicochemical characteristics might be challenging [70,71,72,73].

Respirable dustiness mass-fractions obtained by the SRD method ranged from <limit of quantification (7 mg kg^−1^) to 156 mg kg^−1^ with TiO_2_ and calcite having the lowest and microspheres and OpTiMat clay having the highest values. The standard deviations were <43%, and as low as 2% for calcined clay PoleStar 200P, which demonstrates a general reproducibility and homogeneity among the three replicas considered in dustiness test and the method itself. According to the EN 15,051 ranking scheme, these materials are in the category of powders with a very low dustiness level (<10 mg kg^−1^), low (between 10 and 50 mg kg^−1^), and moderate dustiness index levels (between 50 and 250 mg kg^−1^).

Overall, the pigment/filler powders used at the paint manufacturer were not identified as NMs by the manufacturers considering current regulation [74]. However, the European Commission also proposes to define a material as a NM when it has a volume specific surface area (VSSA) greater than 60, 40, and 20 m^2^ cm^−3^ for spheres, rods/fibers, and flakes, respectively (EU, 2018). Considering uncertainties, Wohlleben et al. [75] proposed the VSSA trigger thresholds of only 24, 16, and 8 m^2^ cm^−3^ for spheres, rods, and flakes, respectively. Hence, considering the shapes of the current study materials and their VSSA values, only clay PoleStar 200P certainly classifies as NM while only microspheres and dolomite can be excluded as not being NMs. The rest of the materials are potentially NMs according to these parameters.

### 3.2. Emissions and Exposure to Chemicals and Particles

Emissions and worker exposure were here analyzed considering the PS or MS and the material being poured (Table 2). Supplementary Table S1 shows the measured organic compounds, which were mainly solvents and coalescent agents. The measured terpenes (α-pinene, 3-carene, and limonene) were probably fragrances. Texanol and the following compounds were coalescent agents used as additive in waterborne paints. The concentrations were generally highest in the solvent room and lowest in outdoor air. The NF, FF, and personal concentrations were generally of comparable magnitude, except during the production of paint batch #2 where NF concentration was lower. All field blanks were below limit of detection defined as 10 times the signal-to-noise ratio. None of the compounds with Danish TLVs exceeded these values (Table S1). For the glycol ethers the highest measured concentrations were from <1 to 14% of the TLV. For white spirit this value was 65%. The results may be regarded as inaccurate due to the calibration with decane since response factors in GC–MS varied significantly. The air concentration may also be somewhat underestimated due to some degree of breakthrough (loss of analyte during sampling) caused by large sampling volumes. However, these results are still deemed useful as an indication of the actual concentrations.

#### 3.2.1. Pouring Activity at the Mixing Station

Particle measurements performed at the MS during non-working hours and specific pouring activities are summarized in Table 2.

During non-working periods (14 h measurements starting on Sunday 28 January at 16 h to Monday at 6 h), the mean particle number and LDSA concentrations measured in the MS at NF were 4800 ± 3000 cm^−3^ and 35.7 ± 21.8 µm^2^ cm^−3^, respectively, and the particle sizes were <200 nm (Supplementary Figure S3). The average NF and FF respirable mass concentrations were 76 and 25 µg m^−3^, respectively (Table 2 and Supplementary Figure S3). Results from electron microscopy analysis showed that the background particles consisted mainly of soot and few pigment/filler particles with vapors condensed on them (Supplementary Figure S4). The daily mean BC was 556 ± 109 and 366 ± 107 ng m^−3^ measured by the BC AE33 and BC_ABCD_ sensors, respectively and data from the two instruments correlated well (R^2^ = 0.82; time series shown in Supplementary Figure S5). The highest BC concentrations were observed between 6:00 and 12:00 CET and similarly, but at lower levels in the afternoon and evening hours reflecting daily variation in traffic. This indicates that particles infiltrated from the outdoor environment. Indoor BC sources were not identified during this measurement campaign.

**Figure 2:**
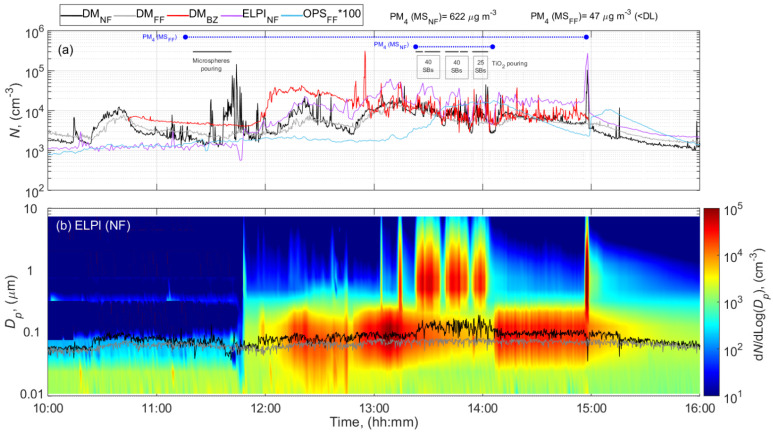
Time series during pouring activities involved in the paint formulation of batch #3 at the MS of (**a**) total particle number concentration, and (**b**) particle number size distributions measured by electrical low-pressure impactor (ELPI) at NF and mean particle size diameter measured by DM at NF and FF. The blue horizontal lines show the filter sampling periods.

Measurements during work hours resulted in considerable episodic increased particle concentrations in all batch formulations. Figure 2 shows an example of the MS NF, FF, and BZ total particle number concentrations and particle number size distributions measured while pouring microspheres (Expancel) and 105 SBs of TiO_2_ during formulation of paint batch #3. The color plot displayed in Figure 2b shows the particle diameter on the y axis, and time of the day on the x axis, with the particle number concentration expressed as d*N*/dlog*D_p_* in each size interval. The normalized concentration d*N*/dlog*D_p_* corresponds to the differential number of particles per differential log diameter within an interval of the size distribution where *N* is the particle number and *D_p_* is the particle diameter.

Pouring of the same powders and clay OpTiMat during formulation of different paint batches, resulted in similar average work day concentrations (Table 2 and Supplementary Figures S6–S9) with higher episodically increased dust concentrations in the NF and BZ as compared with the dust concentrations in the BG (Figure 2, and Table 2). However, increased particle concentrations were also seen in the FF as a result of powder handling activities.

The time-series data showed generally similar trends in BZ and NF measurements with the DM, but NF measurements showed relatively high fluctuations during pouring of microspheres while BZ measurements had higher concentrations and fluctuations during pouring of TiO_2_. A general increase is observed in the NF and BZ particle number concentrations between ca. 12:00 and ca. 13:20, which was probably attributed to other sources (not identified) during the lunch break. The NF DM and ELPI data, showed a remarkable difference in the concentrations and variations between ca. 11:00 and 12:00 including the period with pouring of microspheres (Figure 2). This difference may be explained by the presence of particles with modal diameters larger than 300 nm (particle size distributions shown are shown in Supplementary Figure S10). Although the DM has an inlet separator with a 0.7 μm *d_50_* value, some larger particles can still penetrate and dominate the electrometer signals and result in erroneous particle number concentrations [55,76]. However, for some unknown reason, this difference was not registered in the BZ DM measurements.

Even though the particle number concentrations were similar, the highest level of respirable mass concentration was registered at NF while pouring 105 SBs of TiO_2_ (*PM_4_* = 622 µg m^−3^; Table 2 and Figure 2). This suggests that dust mass concentrations were linked to the amount of material used when the pouring rate was similar (60 kg min^−1^). BZ dust concentrations were consistently higher than in the NF (Table 2). This type of observation is not unusual and may be due to several factors, such as closer proximity to the source than the NF measurement during the actual pouring process, influence of other processes, including folding and handling emptied bags, which was done 1 m further away from the NF, and the unknown dominant air flow directions around the MS.

During pouring activities in the MS, the particle size diameter increased when compared to non-activity levels and differences in the particle size distribution patterns among types of materials poured can also be observed. On average, mean emissions were moderately higher and coarser during TiO_2_ pouring and microspheres than clay OpTiMat pouring (mean particle size distributions shown in Figure S10). Microscopic analysis of the samples collected during TiO_2_ pouring activity confirmed the presence of crystalline TiO_2_ in higher number counts (Figure S11a,b). These particles were agglomerated (Figure S11a) having primary particle sizes of 100–500 nm (Figure S11b), which is close to the *d_50_* indicated by the manufacturer (see Table 1). In addition, other micrometer particles such as platelets of talc (Mg_3_Si_4_O_10_(OH)_2_) and kaolinite (Al_2_Si_2_O_5_(OH)_4_) were detected (Figure S11c,d).

#### 3.2.2. Pouring Activity at the Pouring Station

**Figure 3:**
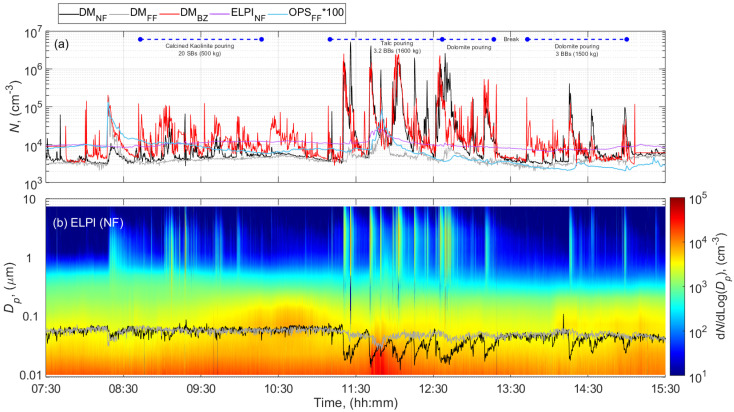
Time series during pouring activities involved in the paint formulation of batch #2 at the PS of (**a**) total particle number concentration, and (**b**) particle number size distributions measured by ELPI at NF and mean particle size diameter measured by DM at NF and FF. The blue horizontal lines show the filter sampling periods.

Figure 3 shows an example of the total particle number concentration and size distribution as a function of time during pouring calcined kaolinite (Ultrex 96), talc (Finntalc M15), and dolomite (Microdol 1) during the paint formulation of batch #2. The measurement results obtained during the formulation of batch #1 and pouring of talc, dolomite, and calcined clay Polestar 200P (Supplementary Figure S12) were comparable to the measurements during formulation of batch #2. The time series of the total particle number concentration and particle size distribution during pouring 332 kg of calcite in the batch #3 paint formulation are illustrated in the Supplementary Figure S13.

The concentrations in terms of particle number, mass and LDSA increased up to 3 orders of magnitude in the NF, BZ, and FF from the preactivity concentration levels during the day (Table 2; Figure 3 and Supplementary Figures S12 and S13). Figure 3, Figures S12b and S13b show that different pouring events coincide with peaks of particle number concentrations at NF in the size range of 300 nm–7 µm. Figure S14 confirms that the mean particle size distributions obtained for each poured material were exclusively different for particles above 300 nm. Hence, the general increase in the total number concentration, mass, and LDSA could be assumed to be due to particles released from powder pouring activities.

Pouring of 925 kg calcined clay and 500 kg kaolinite from SBs, during the formulation of batch #1 and #2, respectively, increased the concentrations >1 × 10^4^ cm^−3^ and 1 × 10^2^ µm^2^ cm^−3^ at NF and BZ (Figure 3 for batch #2 and Supplementary Figure S12 for batch #1) while kept stable at FF location (<4 × 10^3^ cm^−3^ and 10 µm^2^ cm^−3^). However, the NF ELPI concentrations were nearly constant <1 × 10^4^ cm^−3^ and similar to the concentration levels measured before the activity. These differences can probably be attributed to the aerosol measured, which is composed of particles with modal diameters larger than 300 nm (mean particle size distributions shown in Supplementary Figure S14).

**Figure 4:**
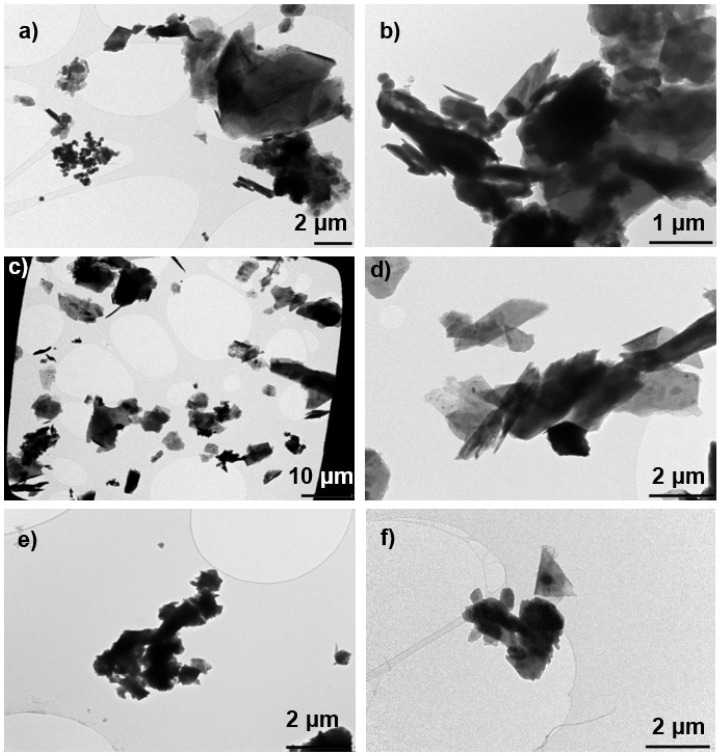
Example of microscope images of particles collected NF during pouring activities of (**a**) calcined clay (PoleStar P200); (**b**) kaolinite (Ultrex 96); (**c**,**d**) talc (Finntalc M15); (**e**) dolomite (Microdol 1); and (**f**) calcite (Socal P2).

The PM_10_ concentrations measured in the NF and in the BZ ranged from 0.6 to 2.1 mg m^−3^ with the highest concentration registered during pouring of kaolinite. Lower PM_10_ mass concentration <0.1 mg m^−3^ were previously measured by Koivisto et al. (2015) at NF while pouring calcined kaolin. This difference between the current and previous concentration measurements is probably due to the low respirable dustiness index of the kaolin material used in the previous study, which was only 30% of the dustiness index of the calcined kaolinite used in the present study or attributed to setup differences. The dust collected during pouring of calcined clay (Figure 4a) and kaolinite (Figure 4b) were dominated by compact and thick Al-Si platelets with >100 nm primary particle size. Some of the collected fragments seemed to be fiber-like particles with a diameter smaller than 3 µm, a length larger than 5 µm and an aspect ratio (length:diameter; L:D) greater than or equal to 3:1, thus meeting the criteria for a respirable fiber according to the World Health Organization definition [77].

Pouring of talc from BBs resulted into the highest concentrations (>limit of detection 1 × 10^6^ cm^−3^ and 1 × 10^3^ µm^2^ cm^−3^) at both BZ and NF locations by using the DM whereas kept relatively constant at 1 × 10^4^ cm^−3^ by using ELPI at NF (Figure 3 and Supplementary Figure S12). Similar as for the clays and kaolinite pouring activities, these high concentrations are probably due to the presence of coarse particles in the DM air sample as indicated above (Figure S14). In this study, the PM_10_ concentrations measured in the NF and in the BZ were indeed the highest among all the materials considered. It ranged from 2.9 to 4.9 mg m^−3^ at NF and 5.6 to 6.5 mg m^−3^ at BZ. The dust collected contained micrometric platelets (Figure 4c,d). Additionally, among these particles, some appeared to meet the criteria for a respirable fibers according to the WHO definition (L:D > 3:1). A concentration peak was detected at FF between 11:30 and 12:00 (Figure 3), which most likely originate from other activities in the FF of the PS; e.g., driving a forklift, moving sacks, using solvents, rather than from the pouring activities. Lower FF concentrations were expected due to the dilution of the concentrations.

Identical to previous pouring events, pouring of dolomite from BBs (1500 kg in total) also increased the concentrations (>1 × 10^5^ cm^−3^ and 1 × 10^2^ µm^2^ cm^−3^) measured in the NF and BZ by using DM whereas ELPI NF kept concentrations stable at 1 × 10^4^ cm^−3^ (Figure 3 and Supplementary Figure S12). This is again possibly due to the presence of particles with modal diameters >300 nm (Supplementary Figure S14) leading to unreliable DM results.

The PM_10_ concentrations measured in the NF ranged from 0.6 to 1.3 mg m^−3^, and in the BZ ranged from 1.0 to 1.8 mg m^−3^. Koivisto et al. (2015) reported similar levels at NF while pouring dolomite from BBs. Dust collected during this pouring event contained mainly micron-size Mg-Ca particles consisting of >100 nm primary particles (Figure 4e).

Pouring 332 kg of calcite CaCO_3_ during the paint formulation of batch #3 produced a clear number concentration peak at both NF and BZ (elevated from 4 × 10^3^ to 2 × 10^4^ cm^−3^), which was not observed by ELPI at NF or at FF by the DM (Figure S13).

This consistent pattern among most of the pouring activities led us to confirm that pouring calcite is also characterized by high coarse particle concentrations (Supplementary Figure S14), which can lead to untrustworthy DM results. The corresponding PM_10_ concentrations measured in both the NF and BZ were the lowest among all the materials poured (0.3 mg m^−3^), which can be explained by the small particle diameters (mean particle size distributions shown in Supplementary Figure S14) and the low dustiness level. Microscopic analysis confirmed the presence of aggregated particles with spheroidal and elongated shape (Figure 4f).

Similarly to what has been registered in the MS, the exposure measured in the worker’s BZ was consistently higher than the NF concentrations (Table 2).

### 3.3. Comparison of Worker Exposure Concentrations with Recommended Exposure Limits

For the comparison between the exposure concentrations and OEL, it was assumed that workers exposure during pouring activities at the mixing and pouring stations had a daily duration between 2.2 and 5.8 h, and the rest of 8 working hours was spent FF.

Based on chemical identity of the collected particles during each event, a nano reference value (NRV_8h-TWA_) of 4 × 10^4^ cm^−3^ set by Social and Economic Council of the Netherlands for biopersistent granular materials with density < 6 × 10^3^ kg m^−3^ was assigned [78]. For short-term exposures of maximum 15 min (NRV_15min-TWA_), a value of 2 × NRV_8h-TWA_ was used (van Broekhuizen et al. [79]). The NRVs are background-corrected concentrations, which, however, were influenced by source emission events.

The calculated 8 h-TWA exposure *N* concentrations calculated from ELPI (or NS and OPS) were in the range of 3.7 × 10^3^ cm^−3^ and 6.0 × 10^3^ cm^−3^ (Table 3) and hence not exceeding 10 percent of the NRV. Even considering the advised short-term value, none of the pouring materials would have exceeded the NRV, because particle concentrations concentration levels were <1.4 × 10^4^ cm^−3^ (not BG corrected).

In terms of mass concentration, the Danish Working Environment Authority [42] has a PEL for 8-h TWA of 10 mg m^−3^ for total suspended inert mineral dust and TiO_2_. For respirable inert mineral dusts, and clays the 8-h TWA PEL is 5 and 2 mg m^−3^, respectively. The National Institute for Occupational Safety and Health [80] has recommended an exposure limit (REL) of 2.4 mg m^−3^ for fine (respirable; EN 481:1993; ISO 7708:1995) TiO_2_ dust. Considering the Danish 8 h-TWA, the 10 mg m^−3^ PEL for TiO_2_ in total dust, and the 2.4 mg m^−3^ NIOSH REL for fine TiO_2_, it is considered that the TiO_2_ exposure do not exceed any of these legal or recommended values during any working day given that the PM_10_ levels were in the range of 0.3–2.1 mg m^−3^. Even though respirable mass sample was not collected for kaolinite pouring, it is not expected that exposure to respirable mineral clays would have exceeded the PEL of 2 mg m^−3^ because PM_10_ was 2.1 mg m^−3^. Koivisto et al. [35] also showed that personal exposure levels to pigments and fillers were below the current OELs during preparing comparable formulations.

### 3.4. Environmental Release

Outdoor measurements in the facility surroundings (Supplementary Figure S1d) were performed during production activities of three different paint batches. Due to strong winds (85% between 1.4 and 4.4 m s^−1^ and 8% between 4.4 and 8 m s^−1^) during the measurement period and (relatively) and low particulate emissions from the exhaust stacks, no measurable impact from the production activities was found in the vicinity of the plant: exposed samplings and non-exposed samplings show elemental concentrations not significantly different.

Stack emissions were characterized on three days. The exhaust stack (PS or MS), and sampling period was chosen based on the most representative activity in the facility. Therefore, three sampling periods were completed for the pouring stack and one for the mixing stack.

Results from the stack emission connected to the MS (mixing stack) during TiO_2_ pouring activity for the production of paint batch #2 (1475 kg from 59 SBs) revealed an emitted mass rate of 20.3 g ton^−1^ (grams per poured tonne of TiO_2_) in TSP at a concentration of 550 µg m^−3^ (Table 4). Total particle concentrations collected by DGI (i.e PM_2.5_ + particles with a diameter > 2.5 µm) and TSP reference method are not directly comparable due to the known effect of particle losses on walls for DGI [81].

Measured particle sizes by using ELPI in the mixing stack ranged from 50 nm to 1 µm (Supplementary Figure S15). The size distribution given by DGI results (Table 4) shows the same trend with majority of particles (in mass) under 2.5 µm size and a significant proportion below 0.2 µm. Table S2 in the supplementary information contains the elemental concentrations and emission factors measured in TSP and in PM_2.5_, PM_1_, PM_0.5_, and PM_0.2_ fractions obtained by the DGI impactor for the mixing stack. The relevant chemical elements found were Si at a concentration of 63 µg m^−3^, Ti at 28.7 µg m^−3^ (TiO_2_ at 46.7 µg m^−3^), Mg at 28 µg m^−3^, Ca at 25 µg m^−3^, and Al at 26 µg m^−3^. Knowing these parameters it was possible to estimate a TiO_2_ mass emission factor of 0.51 g h^−1^, an amount of 1.7 mg TiO_2_ released per kg of TiO_2_ poured, and a total amount of 0.9 kg of TiO_2_ released per year to outdoors (assuming annual TiO_2_ consumption of 500 ton).

The average emission factor measured in the stack emission from PS (pouring stack) during 3 paint batch productions was 11.3 g ton^−1^ in TSP at a concentration of 4–9 mg m^−3^ (Table 4). Measured particle sizes by using ELPI were generally higher and coarser than in the mixing stack varied from 50 nm to 3 µm (Supplementary Figure S16). An average of two assays were collected where the relevant chemical elements found from the TSP filters were Si at a flow of 0.6 g ton^−1^, Ti at a flow of 0.2 g ton^−1^, Mg at an emission factor of 1.3 g ton^−1^, Ca at flow of 0.2 g ton^−1^, and Al at a flow of 0.3 g ton^−1^. Mass concentrations assessed by the use of the Nanobadge were lower than those measured in the quantitative method by a factor 1.5–3.

Sampling on TEM grids allowed us to obtain microscope images of particles collected in the stack emissions during pouring of TiO_2_ and calcined clay. Examples images are shown in Supplementary Figure S17. Overall, pouring of pigments or fillers was always visible in the samples collected in the stack emissions. The pigment/filler poured or mixed at the time of sampling is the main component visible, but other products can also be detected due to historical cumulative effects (Supplementary Figure S17). Part of TiO_2_ (fraction not available) is in the nanometric range but other products are rather in the micrometric size range.

## 4. Discussion

In this study, a field campaign was conducted at a paint manufacturer, during production of different paint batches. Work tasks with potential impacts on human health and environment such as handling solvents and pouring of pigments and fillers were studied in different pouring lines. The measurements included real time particle monitoring, sampling of airborne organic compounds, collection of airborne particles in filter and TEM samplers simultaneously at near field (close to the MS and PS), far field, breathing zone (personal), stacks, and outdoor surroundings. Use of a high methodological strength is essential to investigate potential risks on both environment and workers health [45,46]. Furthermore, this study facilitates the link of personal exposure and working tasks such as handling, pouring small or big bags, and other types of activities, which can occur outside the near field stationary position (e.g., driving a forklift, moving sacks, and folding empty bags).

### 4.1. Material Characteristics and Propensity to Dust Release

Considering the shapes of the current study materials and their VSSA-values, calcined clay PoleStar 200P certainly classifies as NM (VSSA of plates > 20 m^2^ cm^−3^) while only microspheres and dolomite can be excluded as not being NMs [74]. The rest of the materials can be possibly defined as NMs considering their shapes and uncertainty associated in the use of the VSSA method.

Respirable dustiness mass fractions obtained for all the powders used for paint manufacturing were found to be very low (<10 mg kg^−1^) to moderate (between 50 and 250 mg kg^−1^), with TiO_2_ and calcite having the lowest and microspheres and clay OpTiMat having the highest values. As expected, the emission patterns from the materials under study do not follow a linear correlation with the primary particle size given by the manufacturer [82]. Here it seems that near spherical materials with *d_50_* > 20 µm have higher potential to release respirable particles (e.g., clay OpTiMat, microspheres) when compared to materials with smaller particle diameters (e.g., dolomite, kaolinite, calcined clay, and calcite). Similar observations were previously reported for micron-sized and nanoscale powders [35,37,70,71,72,73]. This is probably due to the weaker cohesive forces and consequently material dustiness is higher and particles with small diameters are more probable to be released. However, several mechanisms can influence on dustiness through agglomeration and soft bridging between particles in powders [82].

The dustiness level and characteristics of dust release from a powder material depends on their characteristics and properties (e.g., density, particle size distribution, moisture content, and extent of aggregation and agglomeration [35,82,83,84,85,86,87,88,89,90]). Additionally, storage conditions can play a role in the dustiness. Levin et al. [88] studied the effect of storage conditions on different powders by subjecting them to constant load and different RH levels (30, 50, and 70%) over 7 days. They observed that the higher RH—the lower is the dustiness, but with considerable difference in the absolute values for Al_2_O_3_, HNO_3_-stabilized TiO_2_, ZnO, and CeO_2_. This effect is expected to be more pronounced for hydrophilic than for hydrophobic powder materials [83].

Hence, the difference in relative humidity (RH) of the air found in the paint factory (varying from 40 to 43% RH by using a TSI VelociCalc) and the conditioned 50% ± 5% RH used in dustiness testing according to settings for standard testing in EN17199 could result in higher dustiness of the powders in the factory as compared to that measured in the test. This aspect needs to be considered when using dustiness data as source strength for exposure assessment because an underestimation of exposure and risk level is possible if the workplace < 50% RH. However, a more elaborate analysis on the specific materials is required to understand to what extent dustiness of these materials are affected by the slightly lower RH.

### 4.2. Particle Emissions and Impact on Worker Exposure

Overall, particle emissions and consequently worker exposure occurred during pouring of pigment/filler powders. The particle number concentrations monitored by the diffusion chargers DMs at NF and BZ showed statistically significant increase in dust concentrations up to 4.0 × 10^5^ cm^−3^ during powder pouring (Table 2). An increase in concentrations was also seen in the FF, but at lower levels. However, the emission patterns were markedly different between the NF DM and the ELPI measurements for all the pouring events, except for TiO_2_. This led us to confirm that pouring activities involved in this study were characterized by coarse particle concentrations (Supplementary Figures S10 and S14), which were outside of the DM’s measurement range and therefore led to unreliable current in the electrometer stages and consequently particle counting [55]. These observations are also in line with Koivisto et al. [76], which reported a noise signal during tungsten-carbide-cobalt (WCCo) sieving and milling attributed to the presence of particles with modal diameter larger than 300 nm. Under these circumstances, other types of particle monitors (e.g., ELPI, OPS, and particle mass concentration monitors or samplers) should be the preferred choice.

Even though the raw materials used in this factory were generally considered to be conventional non-nano materials, exposure to UFPs (diameter ≤ 100 nm) still occurred as documented by measured particle size distributions (Supplementary Figures S10 and S14). UFP were detected during TiO_2_ pouring, and slightly in OpTiMat clay pouring. On the other hand, coarser particles > 300 nm up to 7 µm were released from pouring of other filler materials. Microscopy analysis of the samples collected during pouring events at NF also confirms these facts (Figure 4 and Supplementary Figure S11). Here it should be noted that clays in particular are highly anisotropic sheets, where typically only the sheet thickness is in the nanoscale.

Obviously, BG concentrations dominated by nanoscale particles should not be neglected. Microscopy samples from BG confirmed soot agglomerates infiltrated from outdoors and pigment/filler particles with vapors condensed on them (Supplementary Figure S4) with particle size diameters varying from 50 to 200 nm (Supplementary Figure S4). The BC time series from Supplementary Figure S5a confirmed that indoor BC concentration were less than 20% of common urban background concentrations reported for other European cities (approximately 2000 ng m^−3^ registered Paris, London, Barcelona, and Lugano; [91,92]).

The study conducted by Van Broekhuizen et al. [5] during manufacturing of conventional non-nano waterborne paint, measured significant UFPs concentrations in the BZ by using a diffusion charger NanoTracer, which uses the same measurement principle as DM. Pouring conventional pigment grade TiO_2_ and fillers such as CaCO_3_ and talc released ca. 4.4 × 10^4^ cm^−3^, 6.9 × 10^5^ cm^−3^, and 5.7 × 10^5^ cm^−3^, respectively. On the other hand, Van Broekhuizen et al. [5] did not notice airborne release of UFPs during the pouring of nano-TiO_2_ to a nano-enabled paint (task mean ca. 1.5 × 10^3^ cm^−3^).

In this study, personal exposure concentrations were consistently higher than measured from the NF. PM_10_ concentrations in the stationary NF ranged from 0.3 to 4.9 mg m^−3^ whereas personal exposure was consistently higher (0.3–6.5 mg m^−3^). This is likely because the worker is closer to the source than the stationary measurement station during the powder handling events (especially small bags of materials). Alternatively, workers’ exposure could have occurred for example when empty bags were folded outside of the NF volume so that the particle emission was not detected by instruments at NF. It has also previously been reported during pouring processes in a paint factory that personal PM samples exceed the values measured from the stationary measurement point up to 16 times for PM_1_ and 3.9 times for respirable mass [13,35].

Pouring of talc from BBs resulted into the highest personal PM_10_ concentrations among all the materials studied (PM_10_ = 6.5 mg m^−3^). This can be supported by the particle size distributions, which was dominantly coarser >1 µm. On the other hand, pouring of calcite resulted in the lowest PM_10_ among all the materials poured (0.3 mg m^−3^), which can be explained by the low dustiness level.

The pouring events suggested that emission levels are dependent on the amount involved in the pouring activity when pouring rate is similar: The higher the number of pouring from SBs, the higher is the respirable mass concentration (105 TiO_2_ SBs = 622 µg m^−3^ vs. 59 TiO_2_ SBs = 468 µg m^−3^). Similar conclusion was extracted from pouring BBs of talc at a constant pouring rate 18.5 kg min^−1^: personal PM_10_ during pouring 3.2 BBs was 1.3 times higher than 2.4 BBs of talc). Dolomite pouring from BBs also suggested that emission levels were dependent on the pouring rate: pouring of 1500 kg at 13 kg min^−1^ was 1.7 times higher than pouring of 1500 kg at 12 kg min^−1^. This should be carefully considered in occupational exposure assessment modeling [34,93,94]. Another possible exposure determinant, which was not assessed under this study, is the type of process involving SBs or BBs. However, it is foreseen that pouring SBs of a specific material would release higher amounts of particles than BBs because usually they are connected to a pouring funnel, which provides some process enclosure [13]. Besides the source enclosure, the local exhaust ventilation existing at MS and PS are also expected to be an important risk management measure in place to reduce the potential of particle exposure [38,95].

Even though, none of the exposure limits set by the Danish Working Environment Authority (10 mg m^−3^ for total dust) and the National Institute for Occupational Safety and Health (2.4 mg m^−3^ REL for fine TiO_2_) appears to have been exceeded during any of the work situations, additional measurements are advisable to be conducted in order to ensure that workers exposure are systematically lower than 10 percent of the PELs (EN 689:2018 [96]). The compliance with OELs can be guaranteed by increasing the efficacy of the risk management measures. This can be done by improving the technical solutions for process exhaust ventilation. Improvement of work practices and procedures or the powder feeding system could also be implemented in order to minimize particle generation in all the processes. Dust respirators should be used during cleaning procedures as described by NIOSH [97] if technical solutions cannot be achieved.

### 4.3. Environmental Emissions

No measurable impact from the paint production activities were found in the vicinity of the plant due to the low particulate emissions at the stacks and absence of accumulative conditions (i.e., no dispersion with winds < 1 m s^−1^). Particle release to the outdoor environment varied from 6 to 20 g ton^−1^ in TSP at a concentration of 0.6–9.7 mg m^−3^ depending on the powder. Particularly, the estimated amount of TiO_2_ released to outdoors was 0.9 kg per year. Used TiO_2_ and fillers were always detected by TEM analysis in the samples collected at the stack emissions.

Due to the rapid dilution processes, it is not expected that particles released to the environment would generate major environmental and health impacts.

## 5. Conclusions

A field measurement campaign was conducted at a paint manufacturer aiming to quantify workers personal exposures and environmental release of particulate matter during production of different paint batches, which involved pouring of different pigments and fillers.

Airborne particle number concentration (1 × 10^3^–1.0 × 10^4^ cm^−3^), respirable mass (0.06–0.6 mg m^−3^), PM_10_ (0.3–6.5 mg m^−3^), and BC (431–696 ng m^−3^) were measured during pouring processes. The variations in particle concentrations at the near field and breathing zone were found to be dependent on powder pouring rate, and number of repetitions. Emissions from pouring activities were found to be dominated by coarser particles > 300 nm up to 7 µm. Even though the pigment/filler powders used at the paint manufacturer were not identified as NMs by the manufacturers, release of UFPs to workplace air and outdoor environment via the stacks during pouring events, especially for TiO_2_ and clays, was also confirmed. Background nanoscale particles were mostly soot infiltrated from outdoors.

In this study, none of the exposure limits set by the Danish Working Environment Authority for total dust, total TiO_2_, respirable mineral clays, and airborne organic compounds were exceeded during any working day.

An interesting finding from this study was the different responses of the diffusion charger DM when compared to other instruments (e.g., ELPI, OPS, and NanoScan) during pouring processes. It may be concluded that the performance of DMs is not optimal when exposure scenarios are characterized by coarse particles, and therefore that their use for quantification should be discouraged in these cases.

Regarding the environmental release, low emission factor was found at the stacks, depending on the powder (6–20 g ton^−1^ in TSP at a concentration of 0.6–9.7 mg m^−3^). However, no measurable levels were detected in outdoor integrative samplings. Due to the rapid dilution processes, it is not expected that particles released to the environment would generate major impacts.

Overall, the obtained emission source terms will be valuable contribution for occupational and environmental exposure assessment model development and testing. Although no ambient pollution at the site could be detected, the atmospheric modeling could confirm the high level of dilution before the impact of the plume on the ground.

## Figures and Tables

**Table 1:** Description of pouring activities and physicochemical characteristics of the materials under study. σ: standard deviation; *d_50_*: average particle size; SSA: specific surface area; VSSA: volume specific surface area; *DI*: dustiness index; SRD: small rotating drum method; OEL: occupational exposure limit.

Pouring Activity Description				Material Characteristics							OEL ^d^
Material Name	Location	Paint Batch	Measurement Day	*d_50_* (µm) ^¥^	Chemical Composition ^a^	Shape ^a^	Bulk Density ± σ (g cm^−3^) ^b^	BET-SSA ± σ (m^2^ g^−1^)	VSSA (m^2^ cm^−3^)	*DI_SRD_* ± σ (mg kg^−1^) ^c^	(mg m^−3^)
TiO_2_ pigment (93% rutile), (Tioxid TR81; CAS-Nr. 13463-67-7)	MS	#1	30 January 2018	0.25	Major: Ti, O Minor: Si, Al, Zr, P, and organic coating	Sphere	0.94 ± 0.03	12.7 ± 1.3	53.2	3.0 * ± 1.3	6 ^e^
		#2	31 January 2018								
		#3	1 February 2018								
Functionalized alumino-silicate clay (Al_2_Si_2_O_5_, OpTiMat^®^ 2550; CAS No. 93763-70-3)	MS	#1	29 January 2018	25	Major: Si, O Minor: Al, K, Na, and organic coating	Plate	0.16 ± 0.001	5.2 ± 4.0	13.6	149.9 ± 10.8	2
Thermoplastic microspheres (Expancel 461 WE 20 d36; CAS-No. 75-28-5)	MS	#1	29 January 2018	20–30	Organic (2%)	Sphere	N/A	N/A	N/A	155.6 ± 66.3	5 ^f^
		#3	1 February 2018								
Calcined clay (Al_2_Si_2_O_5_; PoleStar™ 200P; CAS No. 92704-41-1)	PS	#1	30 January 2018	2	Major: Si, O, Al Minor: K, Fe	Plate	0.52 ± 0.01	10.4 ± 0.05	27.0	13.3 ± 0.2	2
Calcined kaolinite (Al_2_Si_2_O_5_(OH)_4_; Ultrex 96; CAS No. 92704-41-1)	PS	#2	31 January 2018	0.8	Major: Si, O, Al Minor: Fe, Na, Ti	Plate	0.35 ± 0.01	6.2 ± 0.01	16.0	7.1 ± 0.4	2
Dolomite (CaMg(CO_3_)_2_; Microdol 1; CAS Nr. 16389-88-1)	PS	#1	30 January 2018	7.5	Major: Ca, O, Mg Minor: Si, S	Sphere	1.09 ± 0.04	3.2 ± 0.3	9.7	23.3 ± 1.2	5 ^f^
		#2	31 January 2018								
Talc (Mg_3_Si_4_O_10_(OH)_2_; Finntalc M15; CAS No. 14807-96-6)	PS	#1	30 January 2018	5 (Particles < 2 μm: 20%)	>96% Talc (Mg-Silicate with residue magnesite and chlorite); MgO; SiO_2_; Al_2_O_3_ and FeO)	Plate	0.46 ± 0.01	5.6 ± 0.2	15.1	69.1 ± 4.9	5 ^f^
		#2	31 January 2018								
Calcite (CaCO_3_, Socal^®^ P2, Fine Grades, calcium carbonate >= 98%; CAS No. 471-34-1)	PS	#3	1 February 2018	N/A	Major: Ca, O. Minor: Si, S, Mg	Rod	0.57 ± 0.01	6.7 ± 0.6	18.2	0.6 * ± 0.4	5 ^f^

^a^ Determined by SEM or TEM-EDS; ^b^ Determined according to the procedure given in EN17199-3:2019 [40]; ^c^ Mass-based respirable dustiness determined by small rotating drum (SRD; EN17199-4:2019 [41]); ^d^ Respirable OEL according to the Danish Working Environment Authority [42]; ^e^ Calculated as Ti 8-h time weighted average; ^f^ Respirable inert mineral dust; ^¥^ Information available in the material safety data sheets provided by the manufacturer; * Below the limit of quantification (7 mg kg^−1^); N/A: Not available data.

**Table 2:** Descriptive statistics for the measured particle number concentrations (*N*), and lung deposited surface area (LDSA) by using DiSCmini (DM) (size range 10–700 nm) for non-working hours and for each poured material during each batch production and respirable dust and PM_10_.

Variables		Amount Poured (kg)	Pouring Rate (kg min^−1^)	*N* (cm^−3^)						*LDSA* (µm^2^ cm^−3^)						Respirable Dust/PM_10_ (µg m^−3^)		
				BZ		NF		FF		BZ		NF		FF				
				Mean	±σ	Mean	±σ	Mean	±σ	Mean	±σ	Mean	±σ	Mean	±σ	BZ	NF	FF
**Non-working hours** (14 h of measurements)		-	-	N/A		**4.8 × 10^3^**	**3.0 × 10^3^**	6.7 × 10^3^	3.7 × 10^3^	N/A		35.7	21.8	34.1	22.8	N/A	76/N/A	25/N/A
**TiO_2_ pigment**	Batch #1 (55 min)	2150 kg (86 SBs × 25 kg)	39.1	8.3 × 10^3^	2.6 × 10^4^	1.1 × 10^4^	2.1 × 10^4^	5.8 × 10^3^	3.4 × 10^3^	37.2	55.7	57.6	53.9	22.9	14.3	N/A	1450 **	76.4/N/A
	Batch #2 (24 min)	1475 kg (59 SBs × 25 kg)	61.5	**1.9 × 10^4^**	**1.9 × 10^4^**	1.5 × 10^4^	1.2 × 10^4^	1.0 × 10^4^	2.3 × 10^3^	86.1	68.3	78.5	52.1	40.7	15.5	N/A	467.9/1150 **	60.3/N/A
	Batch #3 (42 min)	2625 kg (105 SBs × 25 kg)	62.5	9.6 × 10^3^	1.1 × 10^4^	9.2 × 10^3^	1.1 × 10^4^	6.7 × 10^3^	2.0 × 10^3^	53.8	39.1	65.3	45.3	27.6	11.5	N/A/1624 *	621.8/1060 **	47.3 (<DL)
**Functionalized alumino-silicate clay (Al_2_Si_2_O_5_, OpTiMat^®^ 2550)**	Batch #1 (14 min)	N/A	N/A	N/A		5.3 × 10^3^	9.2 × 10^3^	6.4 × 10^3^	4.4 × 10^4^	N/A		28.0	36.3	12.2	37.3	N/A	57.6/482 **	34.1 (<DL)
**Microspheres (Expancel)**	Batch #1 (17 min)	N/A	N/A	N/A		**3.2 × 10^4^**	**4.6 × 10^4^**	1.6 × 10^3^	6.0 × 10^2^	N/A		57.7	77.0	4.2	1.5	N/A	457.7/1050 **	N/A
	Batch #3 (21 min)	N/A	N/A	**4.5 × 10^3^**	**3.1 × 10^2^**	**4.1 × 10^3^**	**5.5 × 10^3^**	2.4 × 10^3^	3.8 × 10^2^	11.6	0.4	12.4	14.3	8.2	2.52	N/A	N/A	47.3 (<DL)
**Calcined clay (PoleStar™ 200P)**	Batch #1 (75 min)	925 kg (37 SBs × 25 kg)	12.3	**6.3 × 10^4^**	**4.5 × 10^5^**	**1.1 × 10^4^**	**1.9 × 10^4^**	3.4 × 10^3^	9.6 × 10^2^	61.7	221.4	19.3	0.2	6.3	0.01	N/A/1120 *	N/A/630 *	N/A
**Calcined kaolinite (Ultrex 96)**	Batch #2 (94 min)	500 kg (20 SBs × 25 kg)	5.3	**1.7 × 10^4^**	**2.4 × 10^4^**	**6.4 × 10^3^**	**7.3 × 10^3^**	4.0 × 10^3^	5.6 × 10^2^	44.2	49.7	22.0	0.2	14.4	0.01	N/A/2140 *	N/A/650 *	N/A
**Dolomite (Microdol 1)**	Batch #1 (122 min)	1500 kg (3 BBs × 500 kg)	12.3	**3.6 × 10^4^**	**1.3 × 10^5^**	**2.8 × 10^4^**	**8.2 × 10^4^**	4.8 × 10^3^	2.3 × 10^3^	63.5	187.2	33.5	0.7	9.1	0.03	N/A/1040 *	N/A/610 *	N/A
	Batch #2 (117 min)	1500 kg (3 BBs × 500 kg)	12.8	**2.3 × 10^4^**	**6.4 × 10^4^**	**5.0 × 10^4^**	**2.1 × 10^5^**	3.9 × 10^3^	1.3 × 10^3^	44.2	90.5	56.5	1.7	11.2	0.03	N/A/1750 *	N/A/1260 *	N/A
**Talc (Finntalc M15)**	Batch #1 (64 min)	1200 (2.4 BBs × 500)	18.5	**2.7 × 10^5^**	**6.2 × 10^5^**	**4.0 × 10^5^**	**1.1 × 10^6^**	8.1 × 10^3^	6.0 × 10^3^	449.4	981.5	327.6	7.9	13.1	0.08	N/A/5630 *	N/A/4880 *	N/A
	Batch #2 (87 min)	1600 (3.2 BBs × 500)	18.4	**1.9 × 10^5^**	**5.0 × 10^5^**	**1.9 × 10^5^**	**6.3 × 10^5^**	5.9 × 10^3^	3.3 × 10^3^	313.9	810.4	185.6	4.5	16.1	0.06	N/A/6510 *	N/A/2930 *	N/A
**Calcite (Socal^®^ P2)**	Batch #3 (43 min)	333 (13.3 SBs × 25 kg)	7.7	**8.4 × 10^3^**	**5.8 × 10^3^**	**6.9 × 10^3^**	**4.9 × 10^3^**	4.3 × 10^3^	6.4 × 10^2^	21.4	10.4	17.0	0.1	11.1	0.02	N/A/300 *	N/A/299 *	N/A

Particle number concentrations significantly different from BG (FF) according to Equation (1) are shown in underlined bold. Mean ± σ: arithmetic mean and corresponding standard deviation; BZ: breathing zone; NF: Near field; FF: far field; DL: detection limit; SB: small bags; BB: big bags; N/A: Not available data. * Measured PM_10_ mass concentrations by using SidePak (model AM520) at the PS ** Measured PM_10_ mass concentrations by using OPC-N2 at the mixing station (MS).

**Table 3:** Background corrected 8 h-TWA particle number (*N*) and PM_10_ exposure concentrations obtained for each working day, and comparison with the nano-reference value (NRV) and the permissible exposure limit (PEL).

Day	N_8h-TWA_	NRV_8h-TWA_ (cm^−3^)	N_8h-TWA_/	PM_10 8h-TWA_	PEL_8h-TWA_ (mg m^−3^)	PM_10 8h-TWA_/
	(cm^−3^)	[78]	NRV_8h-TWA_	(mg m^−3^)	[42]	PEL_8h-TWA_
Day 1	3.7 × 10^3^		0.1	1.4		0.1
Day 2	6.0 × 10^3^	4.0 × 10^4^	0.2	2.1	10	0.2
Day 3	5.1 × 10^3^		0.1	0.3		0.03

**Table 4:** Emissions detected at the stack of both mixing and pouring stations in terms of the total particle number, total suspended particles (TSP), and size fraction particle mass.

Variables		ELPI	DGI Sampler					TSP Reference Method [66]
		N total × 10^3^ (cm^−3^)	PM_2.5_ + >2.5 µm (mg m^−3^)	PM_2.5_ (mg m^−3^)	PM_1_ (mg m^−3^)	PM_0.5_ (mg m^−3^)	PM_0.2_ (mg m^−3^)	(mg m^−3^)
Mixing station	Concentration batch #2 (7:33–12:43; TiO_2_ pouring)	2.69	0.38	0.34	0.28	0.18	0.16	0.55
	Flow (g h^−1^/g ton^−1^)	-	4.0/14	3.7/13	3.0/10.5	2.0/7.0	1.7/6.0	5.8/20.3
Pouring station	Concentration batch #1 (7:30–12:32)	4.78	*	*	*	*	*	9.67
	Concentration batch #2 (13:00–14:40)	5.15	2.85	1.87	0.89	0.47	0.26	-
	Concentration batch #3 (12:13–14:57)	9.13	3.49	2.29	1.07	0.44	0.30	4.19
	Average	6.35	3.17	2.08	0.98	0.46	0.28	6.93
	Flow batch #1 (g h^−1^/g ton^−1^)	-	-	-	-	-	-	12.0/16.7
	Flow batch #2 (g h^−1^/g ton^−1^)	-	3.6/4.0	2.3/2.6	1.1/1.2	0.6/0.7	0.3/0.3	5.2/5.8
	Flow batch #3 (g h^−1^/g ton^−1^)	-	4.4/23	2.9/15.2	1.3/6.8	0.6/3.1	0.4/2.1	-
	Average (g h^−1^/g ton^−1^)	-	4.0/13.5	2.6/8.9	1.2/4.0	0.6/1.9	0.4/1.2	8.6/11.3

* DGI spectrum distribution not estimated due to the saturated sample.

## Data Availability

The data presented in this study are available on request from the corresponding author.

## Supplementary Materials

The following are available online at https://www.mdpi.com/1660-4601/18/2/418/s1. Figure S1. Pictures of the indoor and outdoor measurement sites; Figure S2. Transmission electron microscopy images of: (a) TiO_2_ pigment (Tioxid TR81); (b) functionalized Al_2_Si_2_O_5_ (OpTiMat^®^ 2550); (c) Microspheres (Expancel); (d) calcined clay (PoleStar™ 200P); (e) Calcined kaolinite (Ultrex 96); (f) Dolomite (Microdol 1); g) Talc (Finntalc M15); h) calcite, (Socal^®^ P2); Figure S3. Time series during non-working hours of (a) total particle number concentration, and (b) particle number size distributions measured by NanoScan (NS) and optical particle sizer (OPS) at NF and mean particle size diameter measured by DiSCmini (DM) at near field (NF) and far field (FF) in the mixing station. The blue horizontal line show the filter collection time at NF and FF; Figure S4. Example of microscope images of background particles collected in the mixing station during non-working hours; Figure S5. (a) Time series of indoor black carbon (BC) and PM_10_ levels monitored in the mixing station, and (b) Regression analysis for the BC measured with sensor BC ABCD and AE33 based on 1-min resolution data; Figure S6. Time series during pouring alumino-silicate clay (Al_2_Si_2_O_5_, OpTiMat) involved in the paint formulation of batch #1 at the MS of (a) total particle number concentration, and (b) particle number size distributions measured by NanoScan (NS) and OPS at NF and mean particle size diameter measured by DiSCmini (DM) at NF and FF; Figure S7. Time series during pouring microspheres involved in the paint formulation of batch #1 at the MS of (a) total particle number concentration, and (b) particle number size distributions measured by ELPI at NF and mean particle size diameter measured by DiSCmini (DM) at NF and FF. The blue horizontal line shows the filter sampling period; Figure S8. Time series during pouring TiO_2_ involved in the paint formulation of batch #1 at the MS of (a) total particle number concentration, and (b) particle number size distributions measured by ELPI at NF and mean particle size diameter measured by DiSCmini (DM) at NF and FF. The blue horizontal line shows the filter sampling period; Figure S9. Time series during pouring TiO_2_ involved in the paint formulation of batch #2 at the MS of (a) total particle number concentration, and (b) particle number size distributions measured by ELPI at NF and mean particle size diameter measured by DiSCmini (DM) at NF and FF. The blue horizontal line shows the filter sampling period; Figure S10. Averages of near field (NF) particle number size distributions for background (BG), and pouring events at the mixing station; Figure S11. Example of transmission electron microscopy images of particles collected NF during TiO_2_ pouring activities at mixing station; Figure S12. Time series during pouring activities involved in the paint formulation of batch #1 at the PS of (a) total particle number concentration, and (b) particle number size distributions measured by ELPI at NF and mean particle size diameter measured by DM at NF and FF. The blue horizontal line shows the filter sampling period; Figure S13. Time series during pouring CaCO_3_ involved in the paint formulation of batch #3 at the PS of (a) total particle number concentration, and (b) particle number size distributions measured by ELPI at NF and mean particle size diameter measured by DiSCmini (DM) at NF and FF. The blue horizontal line shows the filter sampling period; Figure S14. Averages of near field (NF) particle number size distributions for background (BG), and pouring events at the pouring station; Figure S15. Particle size distribution by ELPI at the mixing exhaust stack; Figure S16. Particle size distribution by ELPI at the pouring stack; Figure S17. Example of transmission electron microscopy images of particles collected in the stack emissions during TiO_2_ and calcined clay pouring; Table S1. Semi-quantitative air concentrations of tentatively identified organic compounds in decane equivalents in different work scenarios; Table S2. Elemental concentrations and emission factors measured in TSP using the reference method and in PM_2.5_, PM_1_, PM_0.5_, PM_0.2_ fractions obtained by the DGI impactor for the mixing stack.

## References

[1] Viitanen A.-K., Uuksulainen S., Koivisto A.J., Hämeri K., Kauppinen T. (2017). Workplace Measurements of Ultrafine Particles—A Literature Review. Ann. Work Expo. Health.

[2] Fonseca A.S., Viana M., Querol X., Moreno N., de Francisco I., Estepa C., de la Fuente G.F., Viana M. (2016). Workplace Exposure to Process-Generated Ultrafine and Nanoparticles in Ceramic Processes Using Laser Technology BT—Indoor and Outdoor Nanoparticles: Determinants of Release and Exposure Scenarios.

[3] Fonseca A.S., Maragkidou A., Viana M., Querol X., Hämeri K., de Francisco I., Estepa C., Borrell C., Lennikov V., de la Fuente G.F. (2015). Process-Generated Nanoparticles from Ceramic Tile Sintering: Emissions, Exposure and Environmental Release. Sci. Total Environ..

[4] Kling K.I., Levin M., Jensen A.C.Ø., Jensen K.A., Koponen I.K. (2016). Size-Resolved Characterization of Particles and Fibers Released during Abrasion of Fiber-Reinforced Composite in a Workplace Influenced by Ambient Background Sources. Aerosol Air Qual. Res..

[5] Van Broekhuizen P., Van Broekhuizen F., Cornelissen R., Reijnders L. (2012). Workplace Exposure to Nanoparticles and the Application of Provisional Nanoreference Values in Times of Uncertain Risks. J. Nanopart. Res..

[6] Buist H.E., Oosterwijk M.T.T. (2017). Applicability of Provisional NRVs to PGNPs and FCNPs. https://www.researchgate.net/profile/Pieter_Broekhuizen/publication/318562315_Applicability_of_provisional_NRVs_to_PGNPs_and_FCNPs/links/597073dba6fdccc6c973a8e6/Applicability-of-provisional-NRVs-to-PGNPs-and-FCNPs.pdf.

[7] Statista (2019). Paint and Coatings Industry—Statistics & Facts. https://www.statista.com/topics/4755/paint-and-coatings-industry/.

[8] The Global Report Painting a Picture of the Industry. www.Akzonobel.com.

[9] Piccinno F., Gottschalk F., Seeger S., Nowack B. (2012). Industrial Production Quantities and Uses of Ten Engineered Nanomaterials in Europe and the World. J. Nanopart.Res..

[10] Schurr G.G., Mark H.E., Othmer D.E., Overberger C.G., Seaborg G., Grayson M. (1981). Paint. Kirk-Othmer Encyclopedia OfChemical Technology.

[11] ILO (2011). Paint and Coating Manufacture. International Labour Organization. https://www.iloencyclopaedia.org/component/k2/item/380-paint-and-coating-manufacture.

[12] US EPA (2008). Sector Performance Report 2008.

[13] Koponen I.K., Koivisto A.J., Jensen K.A. (2015). Worker Exposure and High Time-Resolution Analyses of Process-Related Submicrometre Particle Concentrations at Mixing Stations in Two Paint Factories. Ann. Occup. Hyg..

[14] Møller P., Wallin H. (2000). Genotoxic Hazards of Azo Pigments and Other Colorants Related to 1-Phenylazo-2-Hydroxynaphthalene. Mutat. Res. Mutat. Res..

[15] Saber A.T., Jensen K.A., Jacobsen N.R., Birkedal R., Mikkelsen L., Moller P., Loft S., Wallin H., Vogel U. (2012). Inflammatory and Genotoxic Effects of Nanoparticles Designed for Inclusion in Paints and Lacquers. Nanotoxicology.

[16] Smulders S., Luyts K., Brabants G., Van Landuyt K., Kirschhock C., Smolders E., Golanski L., Vanoirbeek J., Hoet P.H.M. (2014). Toxicity of Nanoparticles Embedded in Paints Compared with Pristine Nanoparticles in Mice. Toxicol. Sci..

[17] Yanamala N., Farcas M.T., Hatfield M.K., Kisin E.R., Kagan V.E., Geraci C.L., Shvedova A.A. (2014). In Vivo Evaluation of the Pulmonary Toxicity of Cellulose Nanocrystals: A Renewable and Sustainable Nanomaterial of the Future. ACS Sustain. Chem. Eng..

[18] Wiemann M., Vennemann A., Wohlleben W. (2020). Lung Toxicity Analysis of Nano-Sized Kaolin and Bentonite: Missing Indications for a Common Grouping. Nanomaterials.

[19] Stone V., Miller M.R., Clift M.J.D., Elder A., Mills N.L., Møller P., Schins R.P.F., Vogel U., Kreyling W.G., Jensen K.A. (2017). Nanomaterials versus Ambient Ultrafine Particles: An Opportunity to Exchange Toxicology Knowledge. Environ. Health Perspect..

[20] EU-OSHA (2009). Workplace Exposure to Nanomaterials.

[21] Gakidou E., Afshin A., Abajobir A.A., Abate K.H., Abbafati C., Abbas K.M., Abd-Allah F., Abdulle A.M., Abera S.F., Aboyans V. (2017). Global, Regional, and National Comparative Risk Assessment of 84 Behavioural, Environmental and Occupational, and Metabolic Risks or Clusters of Risks, 1990–2016: A Systematic Analysis for the Global Burden of Disease Study 2016. Lancet.

[22] Kyung S.Y., Jeong S.H. (2017). Adverse Health Effects of Particulate Matter. J. Korean Med. Assoc..

[23] Landrigan P.J., Fuller R., Acosta N.J.R., Adeyi O., Arnold R., Basu N., Baldé A.B., Bertollini R., Bose-O’Reilly S., Boufford J.I. (2018). The Lancet Commission on Pollution and Health. Lancet.

[24] Lee B.J., Kim B., Lee K. (2014). Air Pollution Exposure and Cardiovascular Disease. Toxicol. Res..

[25] World Health Organization (2016). Ambient Air Pollution: A Global Assessment of Exposure and Burden of Disease.

[26] Health Effects Institute (2018). State of Global Air 2018.

[27] Alleman L.Y., Lamaison L., Perdrix E., Robache A., Galloo J.-C. (2010). PM10 Metal Concentrations and Source Identification Using Positive Matrix Factorization and Wind Sectoring in a French Industrial Zone. Atmos. Res..

[28] Setyan A., Flament P., Locoge N., Deboudt K., Riffault V., Alleman L.Y., Schoemaecker C., Arndt J., Augustin P., Healy R.M. (2019). Investigation on the Near-Field Evolution of Industrial Plumes from Metalworking Activities. Sci. Total Environ..

[29] Buteau S., Shekarrizfard M., Hatzopolou M., Gamache P., Liu L., Smargiassi A. (2020). Air Pollution from Industries and Asthma Onset in Childhood: A Population-Based Birth Cohort Study Using Dispersion Modeling. Environ. Res..

[30] Manisalidis I., Stavropoulou E., Stavropoulos A., Bezirtzoglou E. (2020). Environmental and Health Impacts of Air Pollution: A Review. Front. Public Health.

[31] ECHA (2016). Guidance on Information Requirements and Chemical Safety Assessment Chapter R. 14: Occupational Exposure Assessment.

[32] Kuhlbusch T.A., Asbach C., Fissan H., Göhler D., Stintz M. (2011). Nanoparticle Exposure at Nanotechnology Workplaces: A Review. Part. Fibre Toxicol..

[33] Sørensen S.N., Baun A., Burkard M., Dal Maso M., Foss Hansen S., Harrison S., Hjorth R., Lofts S., Matzke M., Nowack B. (2019). Evaluating Environmental Risk Assessment Models for Nanomaterials According to Requirements along the Product Innovation Stage-Gate Process. Environ. Sci. Nano.

[34] Fransman W., Van Tongeren M., Cherrie J.W., Tischer M., Schneider T., Schinkel J., Kromhout H., Warren N., Goede H., Tielemans E. (2011). Advanced Reach Tool (ART): Development of the Mechanistic Model. Ann. Occup. Hyg..

[35] Koivisto A.J., Jensen A.C.Ø., Levin M., Kling K.I., Maso M.D., Nielsen S.H., Jensen K.A., Koponen I.K. (2015). Testing the near Field/Far Field Model Performance for Prediction of Particulate Matter Emissions in a Paint Factory. Environ. Sci. Process. Impacts.

[36] Koivisto A.J., Kling K.I., Hänninen O., Jayjock M., Löndahl J., Wierzbicka A., Fonseca A.S., Uhrbrand K., Boor B.E., Jiménez A.S. (2019). Source Specific Exposure and Risk Assessment for Indoor Aerosols. Sci. Total Environ..

[37] Ribalta C., Viana M., López-Lilao A., Estupiñá S., Minguillón M.C., Mendoza J., Díaz J., Dahmann D., Monfort E. (2019). On the Relationship between Exposure to Particles and Dustiness during Handling of Powders in Industrial Settings. Ann. Work Exp. Health.

[38] Ribalta C., López-Lilao A., Estupiñá S., Fonseca A.S., Tobías A., García-Cobos A., Minguillón M.C., Monfort E., Viana M. (2019). Health Risk Assessment from Exposure to Particles during Packing in Working Environments. Sci. Total Environ..

[39] Fonseca A.S., Koivisto A.J., Koponen I.K., Jensen A.C.Ø., Kling K.I., Levin M., Mackevica A., Van Tongeren M., Sanchez A., Fransman W. Goodness of Dustiness Index for Predicting Human Exposure to Airborne Nanomaterials. Proceedings of the New Tools and Approaches for Nanomaterial Safety Assessment Conference 2017.

[40] Workplace Exposure—Measurement of Dustiness of Bulk Materials That Contain or Release Respirable Noaa or Other Respirable Particles—Part 3: Continuous Drop Method. https://standards.iteh.ai/catalog/standards/cen/dcbe76ba-cfe9-4e1e-9a4f-3bb891a8e919/en-17199-3-2019.

[41] Workplace Exposure—Measurement of Dustiness of Bulk Materials That Contain or Release Respirable NOAA or Other Respirable Particles—Part 4: Small Rotating Drum Method. https://standards.iteh.ai/catalog/standards/cen/e0179504-3eca-4164-bbab-0cac33ba8c24/en-17199-4-2019.

[42] The Ministry of Employment (2020). Order on Limit Values for Substances and Materials [Danish]. Arbejdstilsynet, j.Nr. 20205200114, Denmark. https://at.dk/regler/bekendtgoerelser/graensevaerdier-stoffer-materialer-698/#Bilag.

[43] Brunauer S., Emmett P.H., Teller E. (1938). Adsorption of Gases in Multimolecular Layers. J. Am. Chem. Soc..

[44] Workplace Exposure—Measurement of Dustiness of Bulk Materials That Contain or Release Respirable NOAA and Other Respirable Particles—Part 1: Requirements and Choice of Test Methods. https://standards.iteh.ai/catalog/standards/cen/0f864553-5d38-4a7a-b613-b310eb5c7a2d/en-17199-1-2019.

[45] OECD (2015). Harmonized Tiered Approach to Measure and Assess the Potential Exposure to Airborne Emissions of Engineered Nano-Objects and Their Agglomerates and Aggregates at Workplaces. ENV/JM/MONO19.

[46] CEN (2018). Workplace Exposure—Assessment of Exposure by Inhalation of Nano-Objects and Their Aggregates and Agglomerates.

[47] Fonseca A.S., Viana M., Pérez N., Alastuey A., Querol X., Kaminski H., Todea A.M., Monz C., Asbach C. (2016). Intercomparison of a Portable and Two Stationary Mobility Particle Sizers for Nanoscale Aerosol Measurements. Aerosol Sci. Technol..

[48] Tritscher T., Beeston M., Zerrath A.F., Elzey S., Krinke T.J., Filimundi E., Bischof O.F. (2013). NanoScan SMPS—A Novel, Portable Nanoparticle Sizing and Counting Instrument. J. Phys. Conf. Ser..

[49] Baron P.A., Willeke K. (2001). Aerosol Measurement Principles, Techniques and Applications.

[50] McMurry P.H. (2002). Chapter 17 A Review of Atmospheric Aerosol Measurements. Dev. Environ. Sci..

[51] (2012). Optical Particle Sizer Model 3330. http://www.tsi.com/uploadedFiles/_Site_Root/Products/Literature/Spec_Sheets/3330_5001323_Web.pdf.

[52] Keskinen J., Pietarinen K., Lehtimäki M. (1992). Electrical Low Pressure Impactor. J. Aerosol Sci..

[53] Matson U., Ekberg L.E., Afshari A. (2004). Measurement of Ultrafine Particles: A Comparison of Two Handheld Condensation Particle Counters. Aerosol Sci. Technol..

[54] (2007). Hand-Held Condensation Particle Counter Model 3007. http://www.tsi.com/uploadedFiles/Product_Information/Literature/Spec_Sheets/3007_1930032.pdf.

[55] Fierz M., Houle C., Steigmeier P., Burtscher H. (2011). Design, Calibration, and Field Performance of a Miniature Diffusion Size Classifier. Aerosol Sci. Technol..

[56] Caubel J.J., Cados T.E., Kirchstetter T.W. (2018). A New Black Carbon Sensor for Dense Air Quality Monitoring Networks. Sensors.

[57] Sousan S., Koehler K., Hallett L., Peters T.M. (2016). Evaluation of the Alphasense Optical Particle Counter (OPC-N2) and the Grimm Portable Aerosol Spectrometer (PAS-1.108). Aerosol Sci. Technol..

[58] Alphasense Ltd. (2015). User Manual: OPC-N2 Optical Particle Counter, 072-0300, Issue 3.

[59] (2016). SIDEPAK^TM^ AM520/AM520i Personal Aerosol Monitor Theory of Operation. Application Note EXPMN-010 Rev. B (10/18/2016). https://tsi.com/getmedia/8b08e494-6ed8-4fab-9c99-9e7ac2688543/EXPMN-010_AM520_Theory_of_Operation-A4-web?ext=.pdf.

[60] (2019). Differences in Mass Measurement Readings When Comparing Photometric Based Instruments. Application Note EXPMN-019 Rev.B (10/7/2019). https://www.tsi.com/getmedia/fc0e33ae-9ea0-444e-9f37-aaba26acda43/EXPMN-019_Photometer_Comparisons_A4-web?ext=.pdf.

[61] Asbach C., Kaminski H., Lamboy Y., Schneiderwind U., Fierz M., Todea A.M. (2016). Silicone Sampling Tubes Can Cause Drastic Artifacts in Measurements with Aerosol Instrumentation Based on Unipolar Diffusion Charging. Aerosol Sci. Technol..

[62] Cheng Y.S., Baron P.A., Willeke K. (2001). Condensation Detection and Diffusion Size Separation Techniques. Aerosol Measurements: Principles, Techniques and Applications.

[63] Mølgaard B., Ondráček J., Švová P., Džumbová L., Barták M., Hussein T., Smolík J. (2014). Migration of Aerosol Particles inside a Two-Zone Apartment with Natural Ventilation: A Multi-Zone Validation of the Multi-Compartment and Size-Resolved Indoor Aerosol Model. Indoor Built Environ..

[64] Pandis S. (2004). Atmospheric Aerosol Processes. Particulate Matter Science for Policy Makers—A Narsto Assessment.

[65] Stacey P., Lee T., Thorpe A., Roberts P., Frost G., Harper M. (2014). Collection Efficiencies of High Flow Rate Personal Respirable Samplers When Measuring Arizona Road Dust and Analysis of Quartz by X-ray Diffraction. Ann. Occup. Hyg..

[66] EN 14385:2004 (2004). Stationary Source Emissions—Determination of the Total Emissions of As, Cd, Cr, Co, Cu, Mn, Ni, Pb, Sb, Ti and V.

[67] R’mili B., Le Bihan O.L.C., Dutouquet C., Aguerre-Charriol O., Frejafon E. (2013). Particle Sampling by TEM Grid Filtration. Aerosol Sci. Technol..

[68] Asbach C., Kuhlbusch T.A.J., Kaminski H., Plitzko S., Götz U., Voetz M., Dahmann D. SOP-Tiered Approach Tiered Approach for the Assessment of Exposure to Airborne Nanoobjects in Workplaces 2012. https://iai-danaserv.iai.kit.edu/files/methodik/SOPs_aus_Projekten/nanoGEM-SOP_tiered-approach-exposure-assessment-workplace_2012.pdf.

[69] Kaminski H., Beyer M., Fissan H., Asbach C., Kuhlbusch T.A.J. (2015). Measurements of Nanoscale TiO_2_ and Al_2_O_3_ in Industrial Workplace Environments? Methodology and Results. Aerosol Air Qual. Res..

[70] López-Lilao A., Escrig A., Orts M.J., Mallol G., Monfort E. (2016). Quartz Dustiness: A Key Factor in Controlling Exposure to Crystalline Silica in the Workplace. J. Occup. Environ. Hyg..

[71] López-Lilao A., Forner V.S., Gasch G.M., Gimeno E.M. (2017). Particle Size Distribution: A Key Factor in Estimating Powder Dustiness. J. Occup. Environ. Hyg..

[72] López-Lilao A., Bruzi M., Sanfélix V., Gozalbo A., Mallol G., Monfort E. (2015). Evaluation of the Dustiness of Different Kaolin Samples. J. Occup. Environ. Hyg..

[73] Pensis I., Mareels J., Dahmann D., Mark D. (2010). Comparative Evaluation of the Dustiness of Industrial Minerals According to European Standard En 15051, 2006. Ann. Occup. Hyg..

[74] European Union (2018). Commission Regulation (EU) 2018/1881 of 3 December 2018 Amending Regulation (EC) No 1907/2006 of the European Parliament and of the Council on the Registration, Evaluation, Authorisation and Restriction of Chemicals (REACH) as Regards Annexes I, III, VI, V. Off. J. Eur. Union.

[75] Wohlleben W., Mielke J., Bianchin A., Ghanem A., Freiberger H., Rauscher H., Gemeinert M., Hodoroaba V.D. (2017). Reliable Nanomaterial Classification of Powders Using the Volume-Specific Surface Area Method. J. Nanopart. Res..

[76] Koivisto A.J., Kling K.I., Levin M., Fransman W., Gosens I., Cassee F.R., Jensen K.A. (2017). First Order Risk Assessment for Nanoparticle Inhalation Exposure during Injection Molding of Polypropylene Composites and Production of Tungsten-Carbide-Cobalt Fine Powder Based upon Pulmonary Inflammation and Surface Area Dose. NanoImpact.

[77] WHO (1997). Determination of Airborne Fibre Number Concentrations.

[78] SER (2012). Provisional Nano Reference Values for Engineered Nanomaterials.

[79] Van Broekhuizen P., Van Veelen W., Streekstra W.H., Schulte P., Reijnders L. (2012). Exposure Limits for Nanoparticles: Report of an International Workshop on Nano Reference Values. Ann. Occup. Hyg..

[80] CDC-NIOSH (2011). Current Intelligence Bulletin 63: Occupational Exposure to Titanium Dioxide.

[81] Ruusunen J., Tapanainen M., Sippula O., Jalava P.I., Lamberg H., Nuutinen K., Tissari J., Ihalainen M., Kuuspalo K., Mäki-Paakkanen J. (2011). A Novel Particle Sampling System for Physico-Chemical and Toxicological Characterization of Emissions. Anal. Bioanal. Chem..

[82] Schneider T., Jensen K.A. (2009). Relevance of Aerosol Dynamics and Dustiness for Personal Exposure to Manufactured Nanoparticles. J. Nanopart. Res..

[83] Shandilya N., Kuijpers E., Tuinman I., Fransman W. (2019). Powder Intrinsic Properties as Dustiness Predictor for an Efficient Exposure Assessment?. Ann. Work Expo. Health.

[84] Chakravarty S., Fischer M., García-Tríñanes P., Parker D., Bihan O.L., Morgeneyer M. (2017). Study of the Particle Motion Induced by a Vortex Shaker. Powder Technol..

[85] Chakravarty S., Le Bihan O., Fischer M., Morgeneyer M. (2017). Dust Generation in Powders: Effect of Particle Size Distribution. EPJ Web Conf..

[86] Jensen K.A., Levin M., Witschger O., Tantra R. (2016). Chapter 10 Methods for Testing Dustiness. Characterization of Nanomaterials: An Introduction.

[87] Le Bihan O.L.C., Ustache A., Bernard D., Aguerre-Chariol O., Morgeneyer M. (2014). Experimental Study of the Aerosolization from a Carbon Nanotube Bulk by a Vortex Shaker. J. Nanomater..

[88] Levin M., Rojas E., Vanhala E., Vippola M., Liguori B., Kling K., Koponen I., Molhave K. (2015). Influence of Relative Humidity and Physical Load during Storage on Dustiness of Inorganic Nanomaterials: Implications for Testing and Risk Assessment. J. Nanopart. Res..

[89] Morgeneyer M., Le Bihan O., Ustache A., Aguerre-Chariol O. (2013). Experimental Study of the Aerosolization of Fine Alumina Particles from Bulk by a Vortex Shaker. Powder Technol..

[90] Jensen K.A., Koponen I.K., Clausen P.A., Schneider T. (2009). Dustiness Behaviour of Loose and Compacted Bentonite and Organoclay Powders: What Is the Difference in Exposure Risk?. J. Nanopart. Res..

[91] Beekmann M., Prévôt A.S.H., Drewnick F., Sciare J., Pandis S.N., Denier van der Gon H.A.C., Crippa M., Freutel F., Poulain L., Ghersi V. (2015). In Situ, Satellite Measurement and Model Evidence on the Dominant Regional Contribution to Fine Particulate Matter Levels in the Paris Megacity. Atmos. Chem. Phys..

[92] Reche C., Querol X., Alastuey A., Viana M., Pey J., Moreno T., Rodríguez S., González Y., Fernández-Camacho R., de la Rosa J. (2011). New Considerations for PM, Black Carbon and Particle Number Concentration for Air Quality Monitoring across Different European Cities. Atmos. Chem. Phys..

[93] Tielemans E., Noy D., Schinkel J., Heussen H., Van Der Schaaf D., West J., Fransman W. (2008). Stoffenmanager Exposure Model: Development of a Quantitative Algorithm. Ann. Occup. Hyg..

[94] Tielemans E., Schneider T., Goede H., Tischer M., Warren N., Kromhout H., Van Tongeren M., Van Hemmen J., Cherrie J.W. (2008). Conceptual Model for Assessment of Inhalation Exposure: Defining Modifying Factors. Ann. Occup. Hyg..

[95] Fonseca A.S., Kuijpers E., Kling K.I., Levin M., Koivisto A.J., Nielsen S.H., Fransman W., Yu Y.F., Antipov A., Jensen K.A. (2018). Particle Release and Control of Worker Exposure during Laboratory-Scale Synthesis, Handling and Simulated Spills of Manufactured Nanomaterials in Fume-Hoods. J. Nanopart. Res..

[96] Workplace Exposure—Measurement of Exposure by Inhalation to Chemical Agents—Strategy for Testing Compliance with Occupational Exposure Limit Values. https://infostore.saiglobal.com/en-us/standards/EN-689-2018-346556_SAIG_CEN_CEN_792425/.

[97] NIOSH (2012). General Safe Practices for Working with Engineered Nanomaterials in Research Laboratories.

