# Supplementary material for: Occupational Exposure and Environmental Release: The Case Study of Pouring TiO2 and Filler Materials for Paint Production
Source: Int J Environ Res Public Health. 2021 Jan 7;18(2):418. doi: 10.3390/ijerph18020418 (PMC7825781; doi:10.3390/ijerph18020418)
Supplement: Supplementary file 1 [file ijerph-18-00418-s001.pdf]

Supplementary material

Occupational exposure and environmental release: The case study of pouring TiO_2 and filler materials for paint production

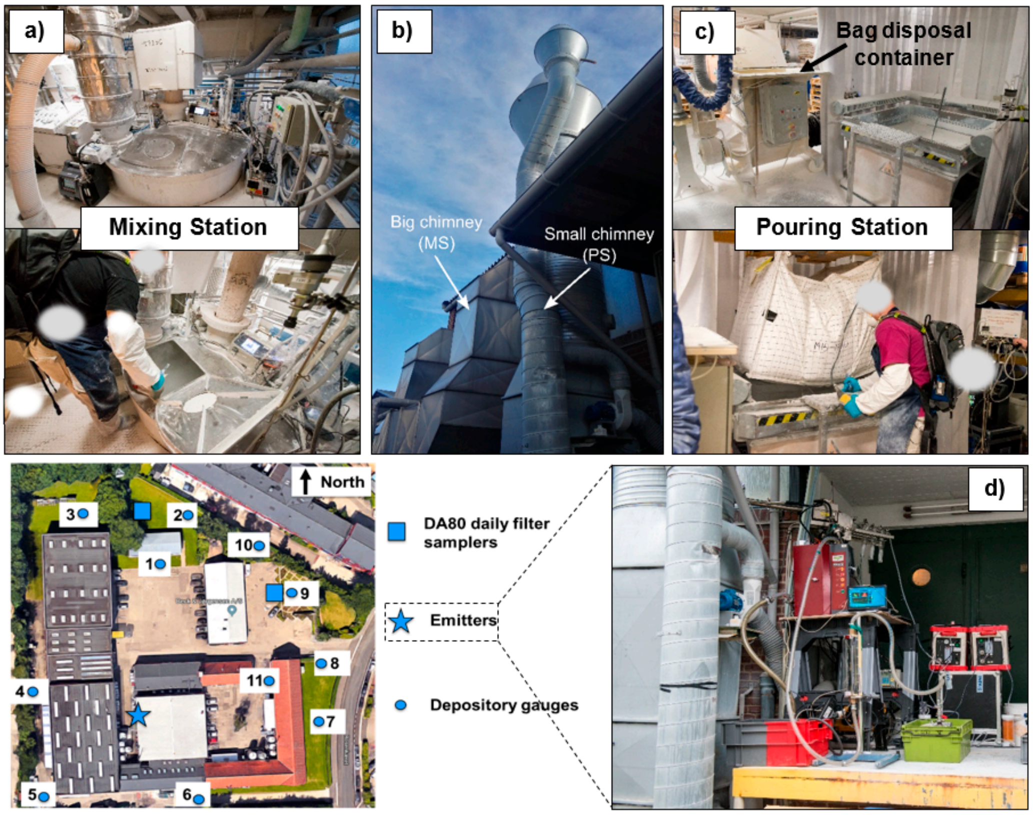

Figure S1. Pictures of the indoor and outdoor measurement sites: a) Mixing station; b) Exhaust stacks; c) Mixing station; and d) environmental and stack emission measurements.

-Methodology followed for environmental monitoring and sampling

- 11 deposition passive gauges have been positioned on the site perimeter. After a sampling period of one month, their content was analyzed by ICP-OES to determine the 1 month averaged particle concentrations in atmospheric deposits.
- 2 high flowrate air daily samplers (DA 80, Digitel, operated at 720 m^3/day) were used. One was positioned off-wind the stacks (considering the observed average wind direction); the second position served as a reference point. One filter was generated by each sampler per day, which were analyzed by ICP-OES, allowing to derive daily mass concentrations of elements in air.
- MPS sampling on TEM grids were used with sampling durations of a few minutes. Several were made at exposed and non-exposed locations. This allows to determine the chemical nature and the morphology of atmospheric particles.
- Nanobadge samples were collected with sampling durations of 1-2 hours. Nanobadge devices are portable instruments that collect airborne particles in the alveolar fraction on PTFE filters. These filters

are subsequently analysed by Total Reflection X-Ray Fluorescence to give semi-quantitative elemental concentrations in airborne particles.

- One portable CPC (TSI model 3007) was used as a control device for total particulate concentration.
- Meteorological station recording wind direction and strength, which allows to determinate, for each fixed sampling devices, the time periods where they have been, or not, exposed to emissions.

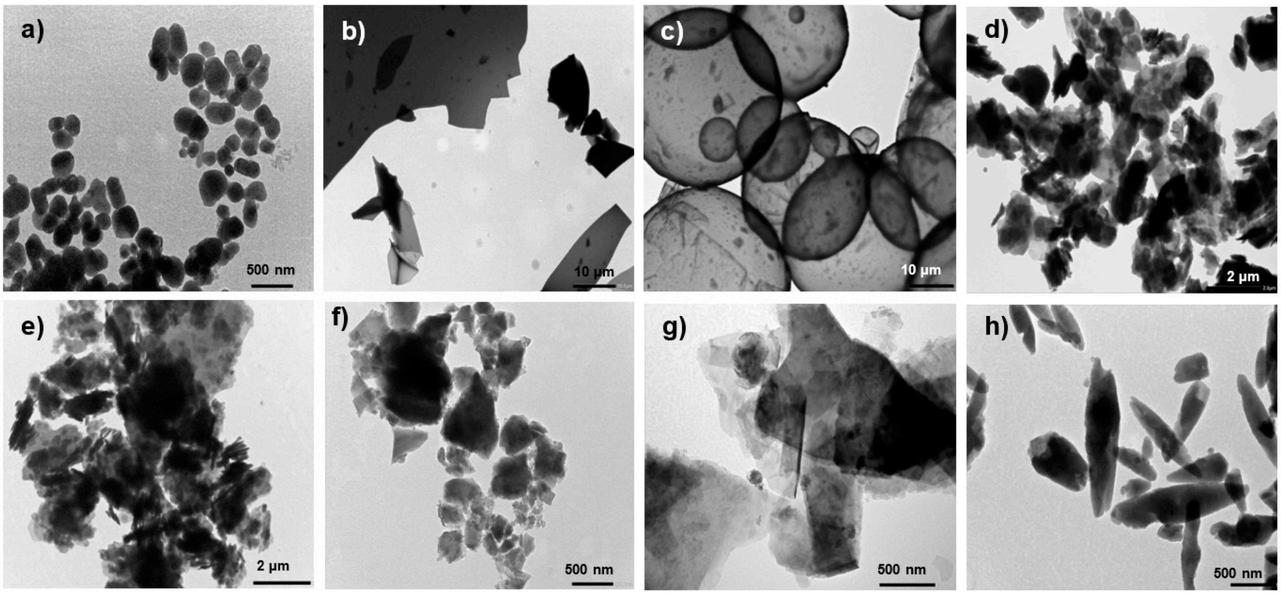

Figure S2. Transmission electron microscopy images of: a) TiO_2 pigment (Tioxid TR81); b) functionalized $Al_2Si_2O_5$ (OpTiMat® 2550); c) Microspheres (Expancel); d) calcined clay (PoleStar™ 200P); e) Calcined kaolinite (Ultrex 96); f) Dolomite (Microdol 1); g) Talc (Finntalc M15); h) calcite, (Socal® P2).

TiO_2 pigment (93% rutile; CAS-Nr. 13463-67-7) is composed of compact crystalline particles with typical individual sizes in the range of 150-300 nm, slightly aggregated (Figure S2a). The functionalized alumino-silicate clay ($Al_2Si_2O_5$, OpTiMat® 2550; CAS No. 93763-70-3) is composed of thin glass-like debris particles (probably amorphous), size in the range 0,2-10 µm (Figure S2b). Microspheres (Expancel 461 WE 20 d36; CAS-Nr. 75-28-5) consist of hollow spheres in the diameter range 5-40 µm (Figure S2c). The calcined clay (PoleStar™ 200P; CAS Nr. 92704-41-1) is composed of thick crystalline platelets with a lateral size range on the order of 1-30 µm. The flakes were often aggregated. Their thickness was around 0,1 µm (Figure S2d). Calcined kaolinite ($Al_2Si_2O_5(OH)_4$; Ultrex 96; CAS Nr. 92704-41-1) is composed of thick crystallized platelets in the lateral size range 0.5-3 µm, strongly aggregated. The flake thicknesses are typically around 0.2 µm (Figure S2e). Dolomite ($CaMg(CO_3)_2$; Microdol 1; CAS Nr. 16389-88-1) is composed of aggregated crystallized platelets with individual lateral size in the range 0,1-5 µm, thickness not visible (Figure S2f). Talc ($Mg_3Si_4O_{10}(OH)_2$; Finntalc M15; CAS Nr. 14807-96-6) is composed of slightly aggregated crystallized platelets with individual lateral size in the range 2-20 µm, thickness not visible (Figure S2g). Lastly, calcite, $CaCO_3$ (Socal® P2, Fine Grades, calcium carbonate >=98%; CAS Nr. 471-34-1) is composed of lenticular/needle-shaped crystalline particles with typical diameters in the size-range of 100-250 nm, strongly aggregated (Figure S2h).

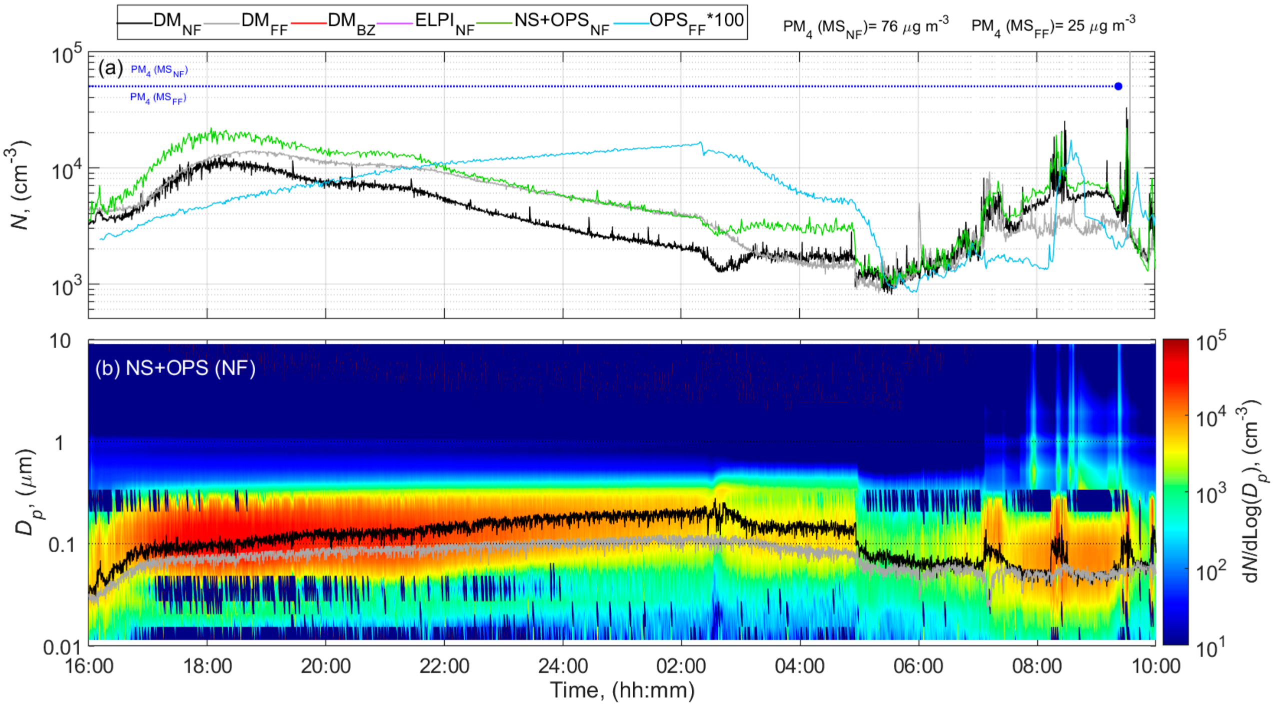

Figure S3. Time series during non-working hours of (a) total particle number concentration, and (b) particle number size distributions measured by NanoScan (NS) and optical particle sizer (OPS) at NF and mean particle size diameter measured by DiSCmini (DM) at near field (NF) and far field (FF) in the mixing station. The blue horizontal line show the filter collection time at NF and FF.

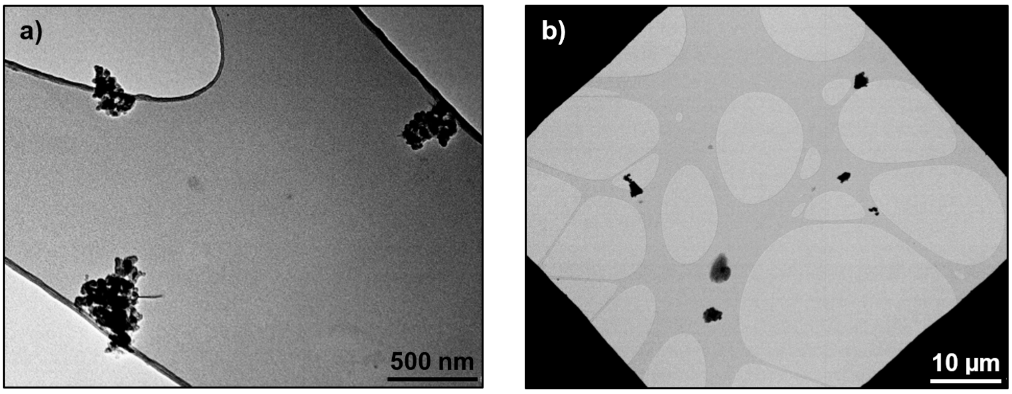

Figure S4. Example of microscope images of background particles collected in the mixing station during non-working hours: a) soot; and b) pigment/filler particles with vapors condensed on them.

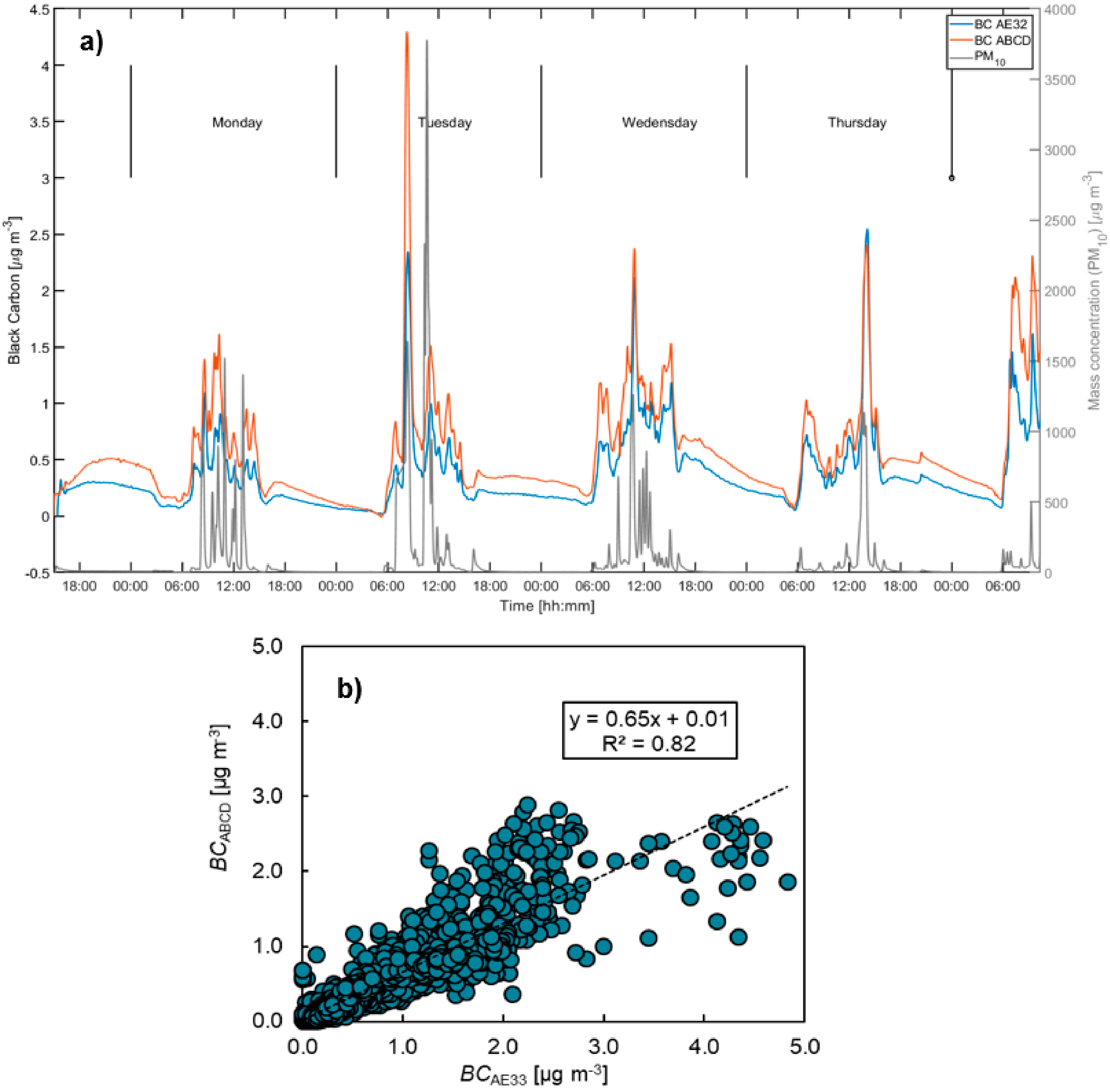

Figure S5. (a) Time series of indoor black carbon (BC) and PM_{10} levels monitored in the mixing station, and (b) Regression analysis for the BC measured with sensor BC ABCD and AE33 based on 1-min resolution data.

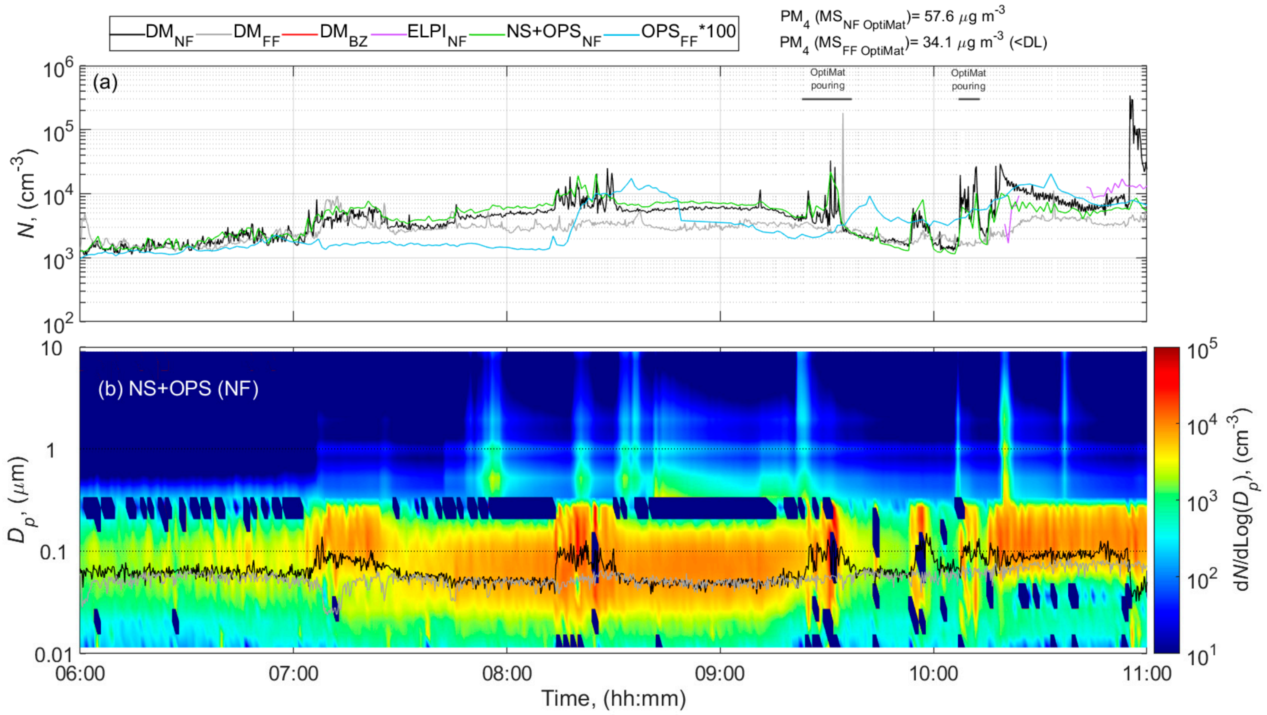

Figure S6. Time series during pouring alumino-silicate clay ($Al_2Si_2O_5$, OpTiMat) involved in the paint formulation of batch #1 at the MS of (a) total particle number concentration, and (b) particle number size distributions measured by NanoScan (NS) and OPS at NF and mean particle size diameter measured by DiSCmini (DM) at NF and FF.

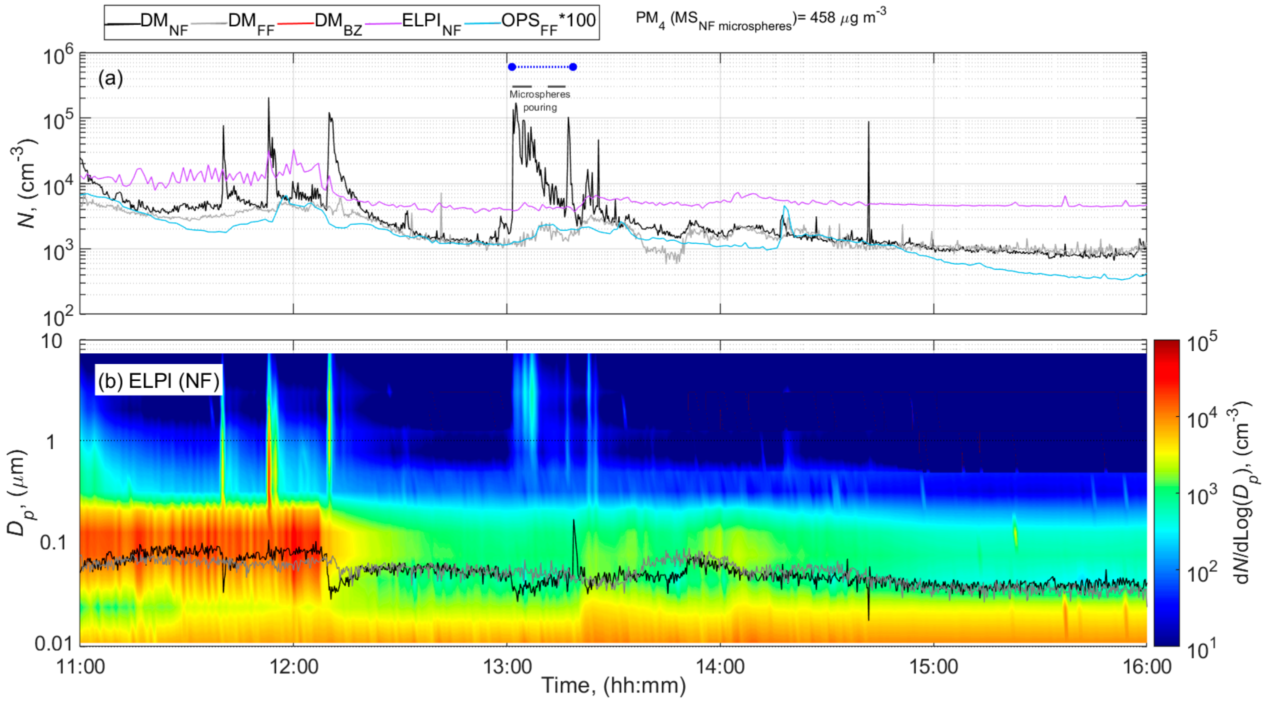

Figure S7. Time series during pouring microspheres involved in the paint formulation of batch #1 at the MS of (a) total particle number concentration, and (b) particle number size distributions measured by ELPI at NF and mean particle size diameter measured by DiSCmini (DM) at NF and FF. The blue horizontal line shows the filter sampling period.

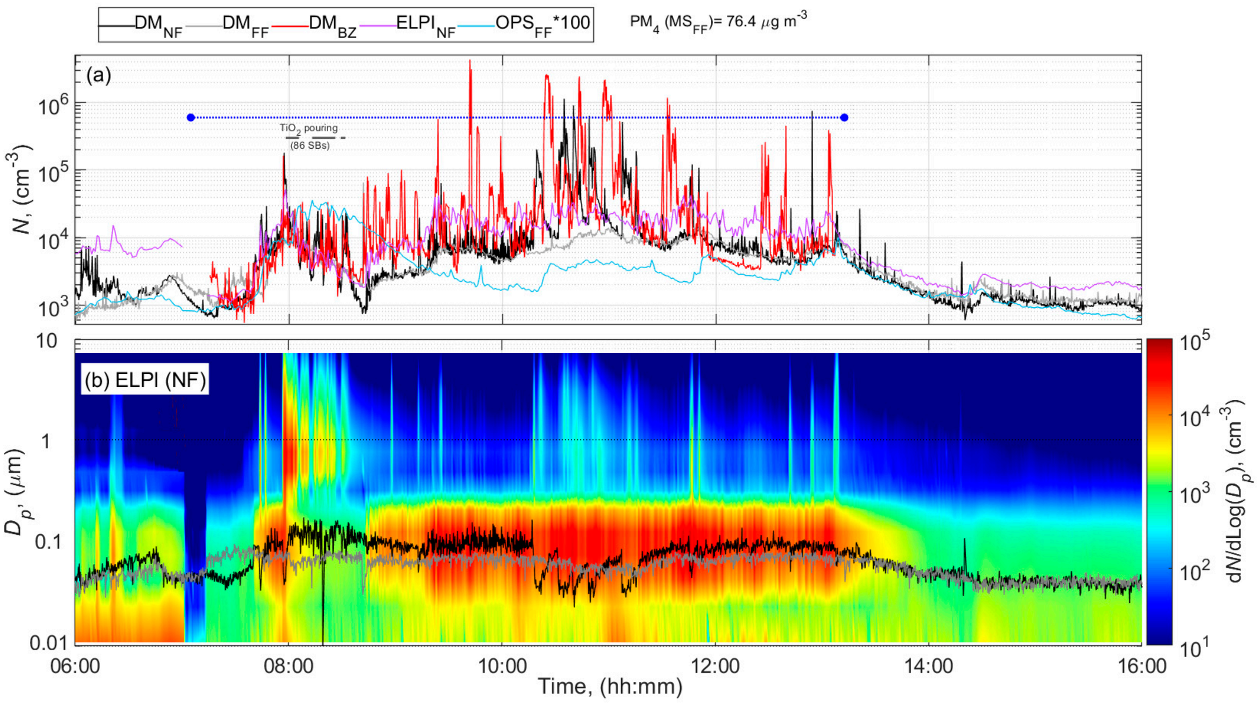

Figure S8. Time series during pouring TiO_2 involved in the paint formulation of batch #1 at the MS of (a) total particle number concentration, and (b) particle number size distributions measured by ELPI at NF and mean particle size diameter measured by DiSCmini (DM) at NF and FF. The blue horizontal line shows the filter sampling period.

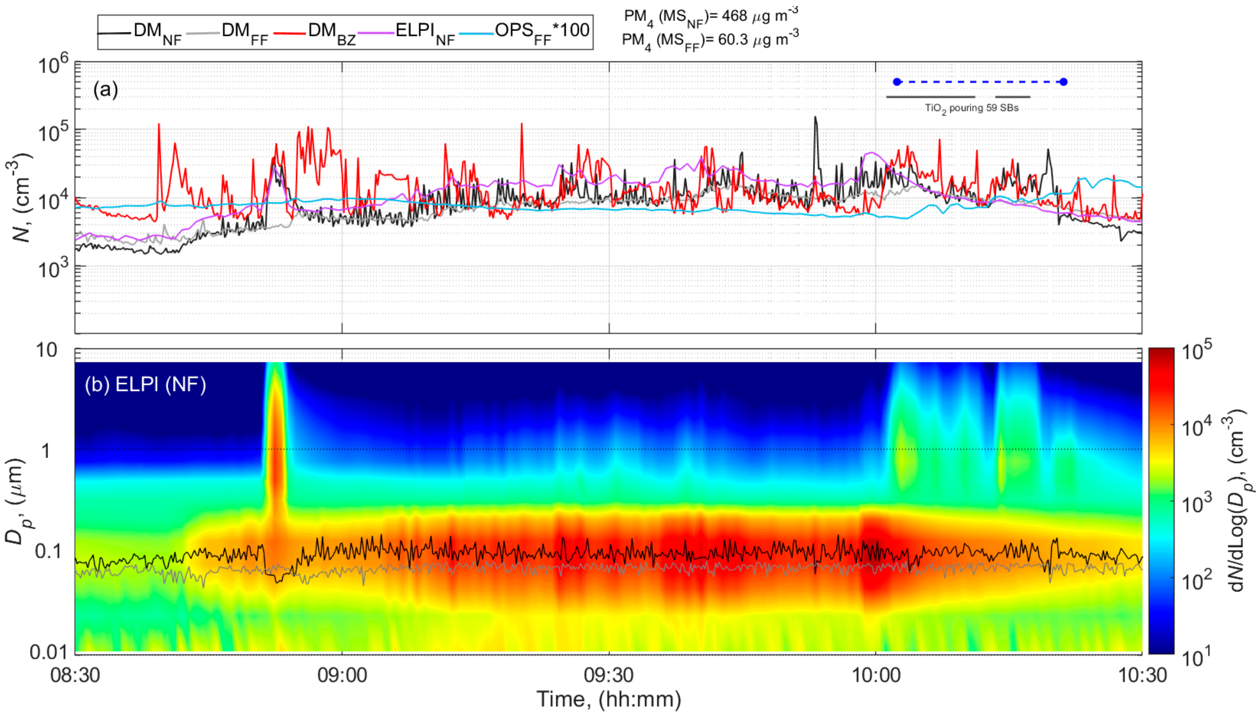

Figure S9. Time series during pouring TiO_2 involved in the paint formulation of batch #2 at the MS of (a) total particle number concentration, and (b) particle number size distributions measured by ELPI at NF and mean particle size diameter measured by DiSCmini (DM) at NF and FF. The blue horizontal line shows the filter sampling period.

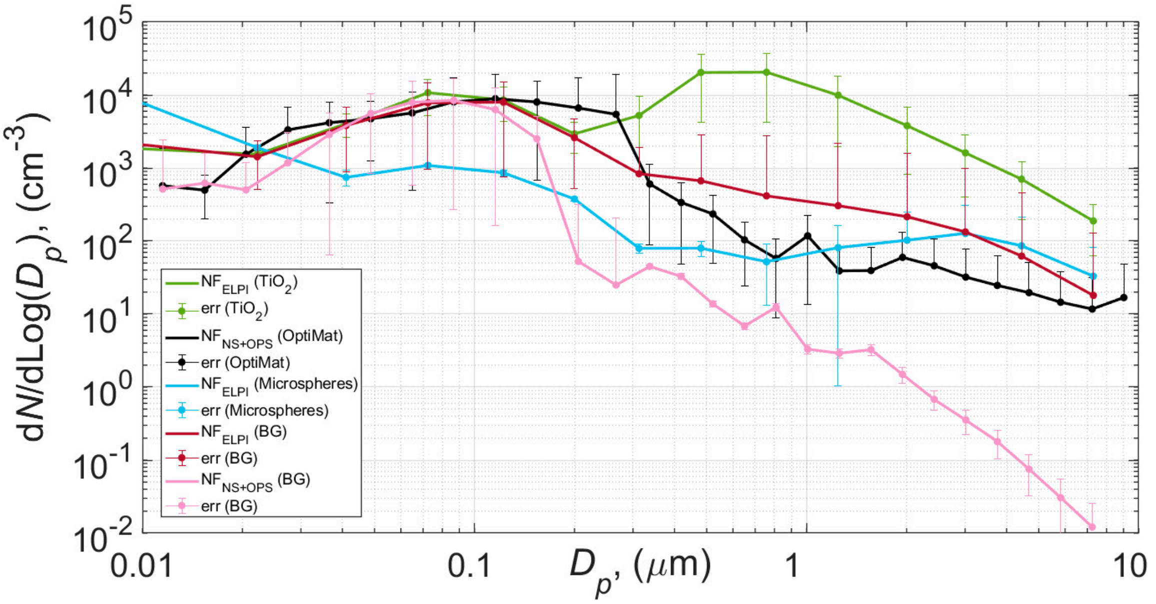

Figure S10. Averages of near field (NF) particle number size distributions for background (BG), and pouring events at the mixing station. The 15 min prior the target activity was used to define the BG concentration.

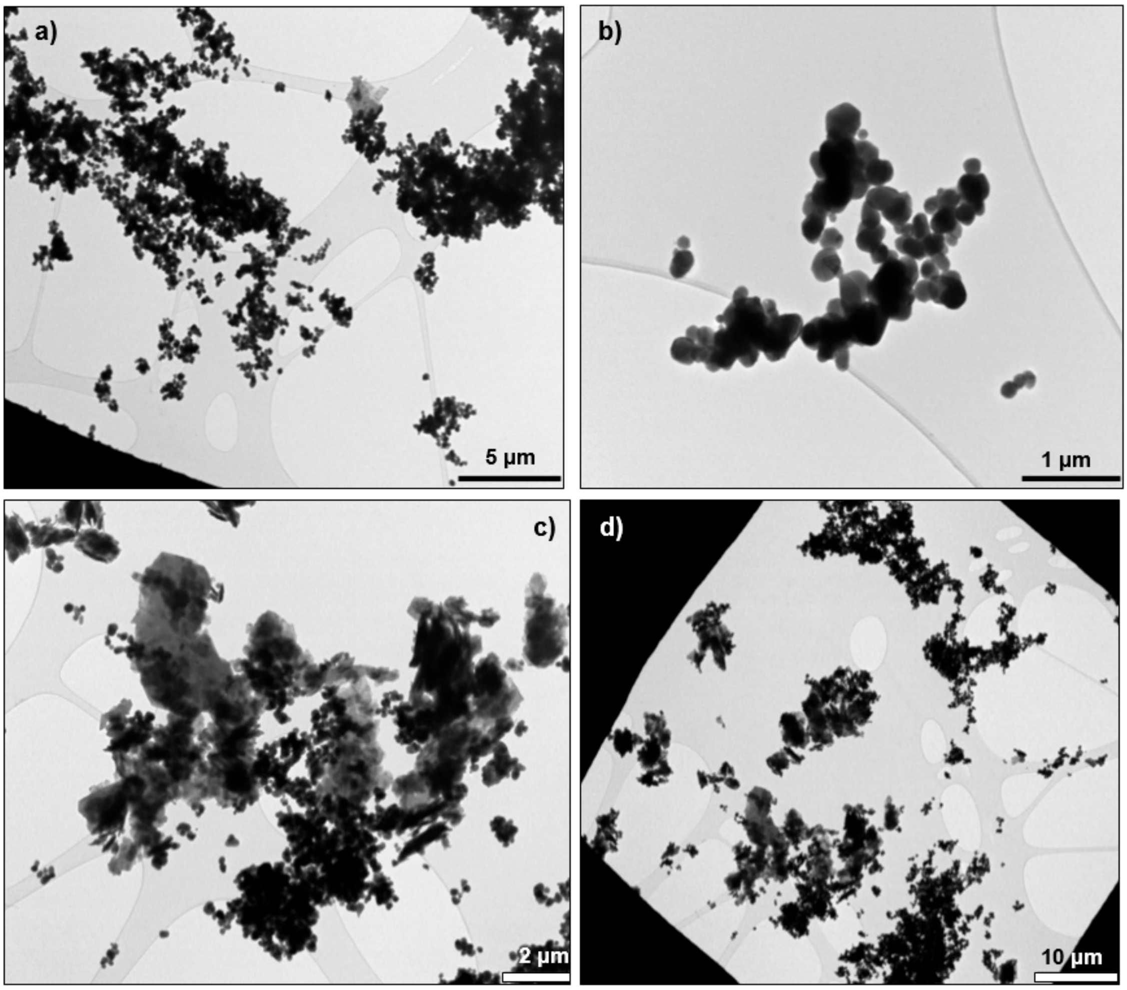

Figure S11. Example of transmission electron microscopy images of particles collected NF during TiO_2 pouring activities at mixing station.

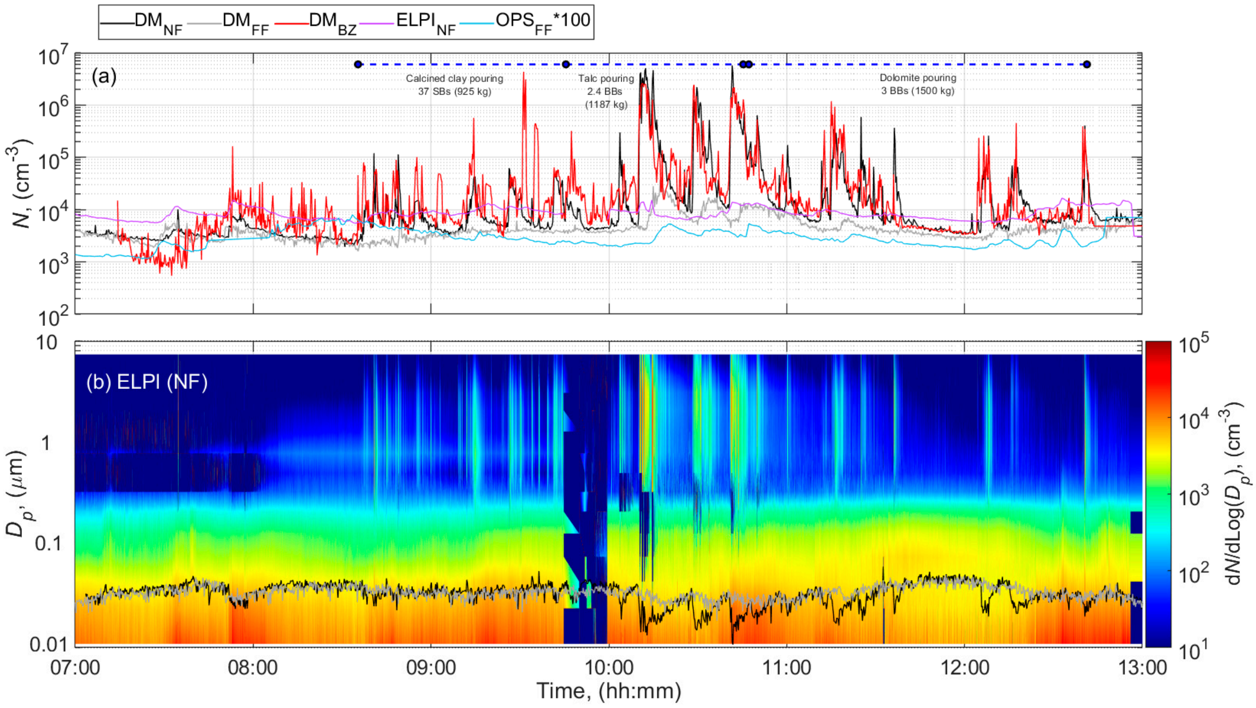

Figure S12. Time series during pouring activities involved in the paint formulation of batch #1 at the PS of (a) total particle number concentration, and (b) particle number size distributions measured by ELPI at NF and mean particle size diameter measured by DM at NF and FF. The blue horizontal line shows the filter sampling period.

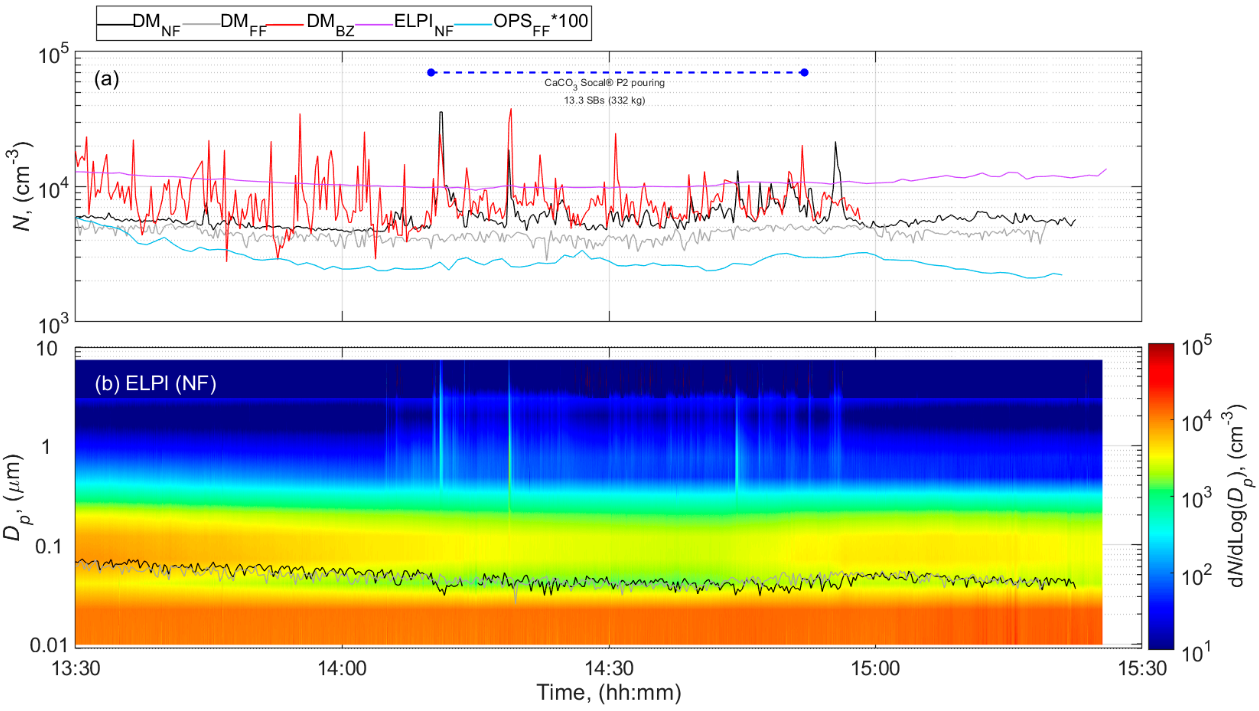

Figure S13. Time series during pouring $CaCO_3$ involved in the paint formulation of batch #3 at the PS of (a) total particle number concentration, and (b) particle number size distributions measured by ELPI at NF and mean particle size diameter measured by DiSCmini (DM) at NF and FF. The blue horizontal line shows the filter sampling period.

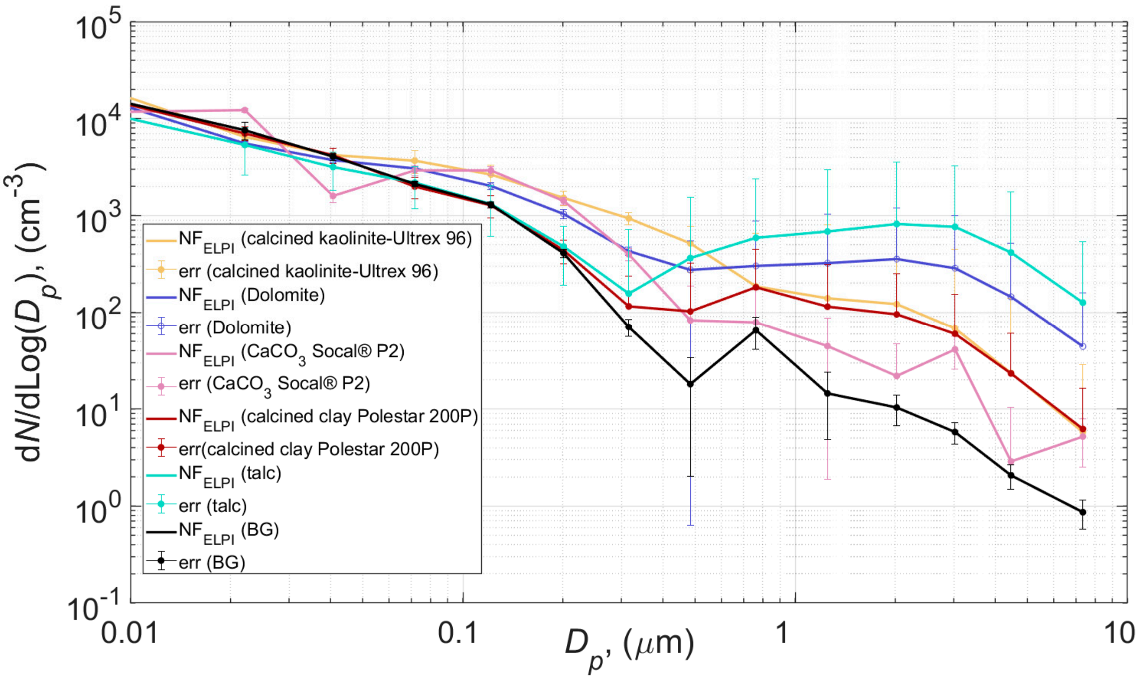

Figure S14. Averages of near field (NF) particle number size distributions for background (BG), and pouring events at the pouring station. The 15 min prior the target activity was used to define the BG concentration.

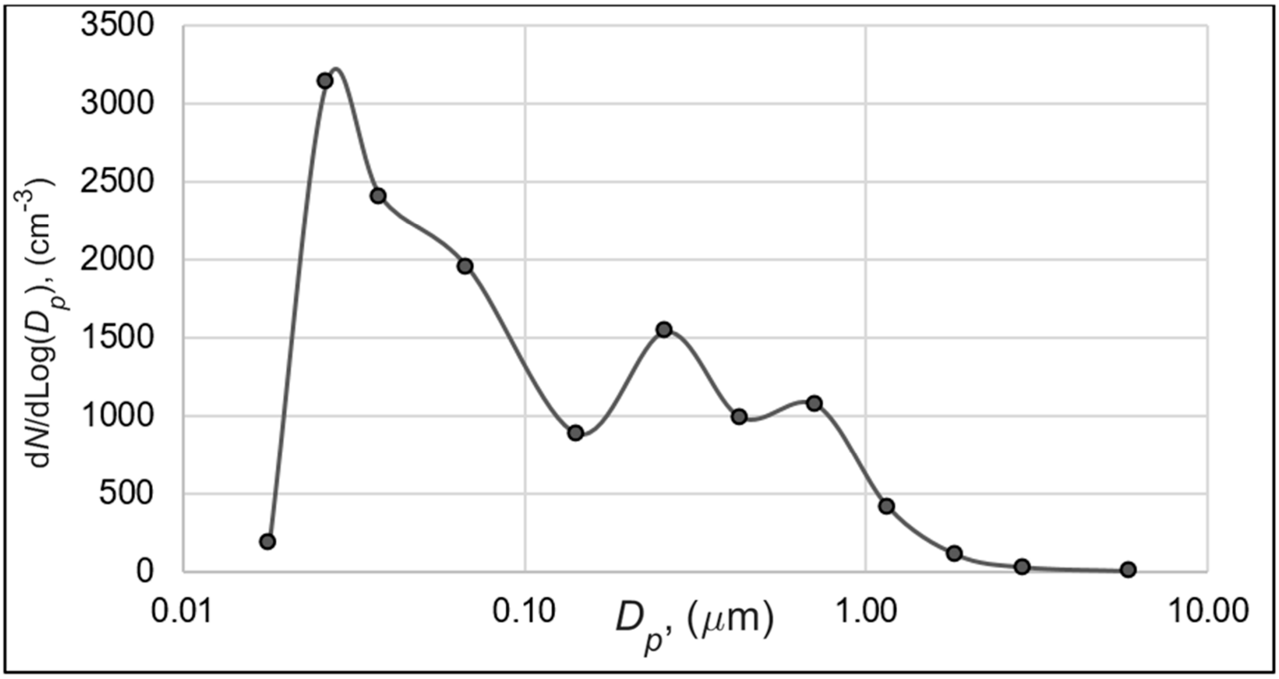

Figure S15. Particle size distribution by ELPI at the mixing exhaust stack.

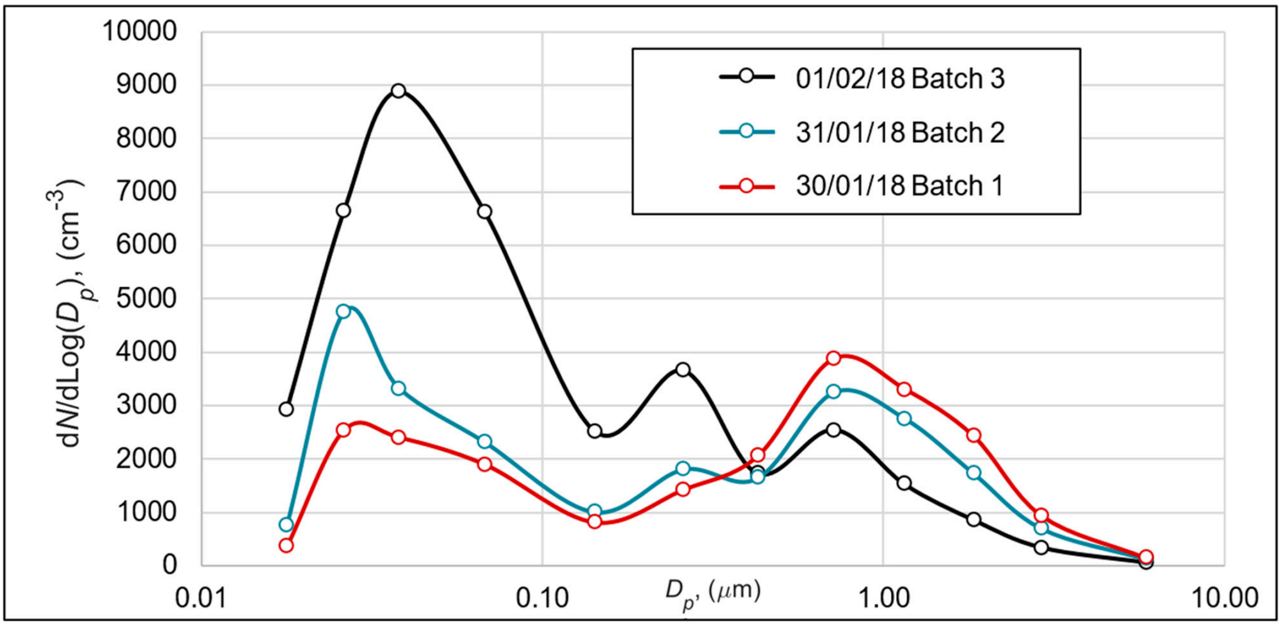

Figure S16. Particle size distribution by ELPI at the pouring stack.

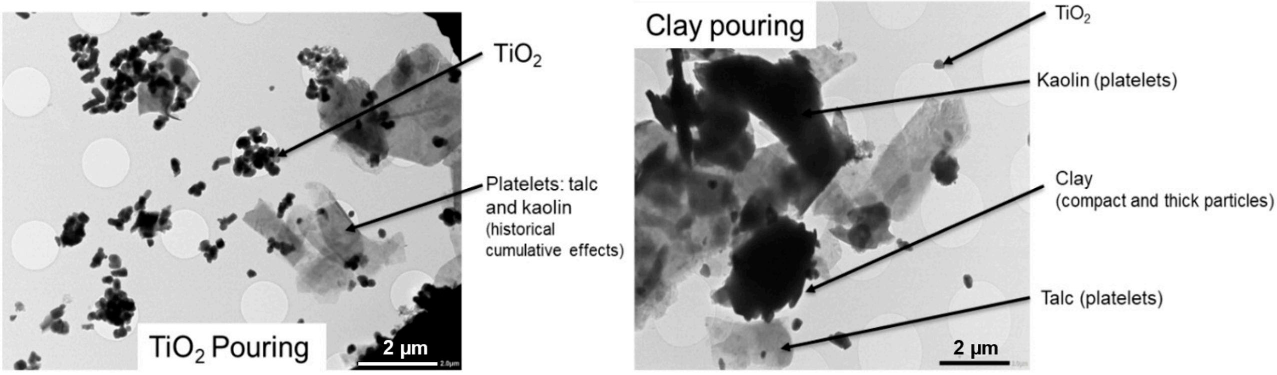

Figure S17. Example of transmission electron microscopy images of particles collected in the stack emissions during TiO_2 and calcined clay pouring.

Table S1. Semi-quantitative air concentrations of tentatively identified organic compounds in decane equivalents in different work scenarios. The organic compounds are listed in the order of GC-MS retention times. PS: Pouring station; NF: Near Field; FF: Far field; BZ: Breathing zone; LOD: Limit of detection defined as 10 times the signal-to-noise ratio; TLV: Threshold limit value. ? = uncertain identification; in all cases this is due to different isomers.

**Measurement location**		**BZ**	**PS FF**	**Solvent room (PS)**	**Outdoor**	**PS NF**	**Field blank**	**BZ**	**PS FF**	**Solvent room PS**	**Outdoor**	**PS NF**	**Field blank**	**Danish TLV ($\mu g\ m^{-3}$)**
**Activity**		**Pouring and mixing**	**Work day activity**	**Work day activity**	**Work day activity**	**Work day activity**		**Pouring and mixing**	**Work day activity**	**Work day activity**	**Work day activity**	**Work day activity**	**-**	
**Date**		Paint batch #2 (31-01-2018)						Paint batch #3 (01-02-2018)						
**Compound***	**GC-MS retention time (min)**	$\mu g\ m^{-3}$	$\mu g\ m^{-3}$	$\mu g\ m^{-3}$	$\mu g\ m^{-3}$	$\mu g\ m^{-3}$	$\mu g\ m^{-3}$	$\mu g\ m^{-3}$	$\mu g\ m^{-3}$	$\mu g\ m^{-3}$	$\mu g\ m^{-3}$	$\mu g\ m^{-3}$	$\mu g\ m^{-3}$	
1,2-propanediol	2.7	1800	1600	2100	< LOD	210	< LOD	2900	1700	80	2	2200	< LOD	
White spirit t_R 4-21 min	4-21	34000	30000	95000		1700	< LOD	53000	41000	61000		41000	< LOD	145000
2-butoxyethanol	5.9	5500	2100	14000	10	280	< LOD	4100	1800	9200	2	2100	< LOD	98000
α-pinene	6.55	350	430	420	2	110	< LOD	910	1100	47	< LOD	840	< LOD	
1-butoxy-2-propanol	7.05	1600	720	11000	< LOD	80	< LOD	1900	580	8000	< LOD	520	< LOD	540700
1-(2-methoxy-1-methylethoxy)-2-propanol ?	9	180	140	3400	< LOD	< LOD	< LOD	600	570	2100	< LOD	970	< LOD	1818400
3-carene	9.14	560	650	620	< LOD	73	< LOD	830	1200	170	< LOD	520	< LOD	
1-(2-methoxy-1-methylethoxy)-2-propanol ?	9.17	110	160	2700	< LOD	< LOD	< LOD	390	430	1500	< LOD	850	< LOD	1818400
Limonene	10.0	86	91	160	< LOD	59	< LOD	140	160	161	0.3	150	< LOD	
2-(2-butoxyethoxy)ethanol	16.7	2700	1700	7700		480	< LOD	2100	2100	160	< LOD	3800	< LOD	67500
Dipropyleneglycol butyl ether	18.4	140	170	230	< LOD	33	< LOD	140	170	12	0.01	25	< LOD	
Dipropyleneglycol butyl ether, isomer of ?	18.6	180	210	340	< LOD	< LOD	< LOD	180	230	< LOD	< LOD	350	< LOD	
Texanol (isomer 1 + 2)	21.9 + 22.3	3000	3400	3800	6	460	< LOD	5500	3400	1300	6	3500	< LOD	
Diisobutyl succinate	23.3	1000	1100	12	13	157	< LOD	930	780	630	2	1100	< LOD	
Glutaric acid, di(isobutyl) ester	23.8	1400	1500	1900	10	400	< LOD	2000	1800	680	2	2200	< LOD	
TXIB	23.9	2100	1700	1900	29	240	< LOD	2100	1300	480	2	1800	< LOD	
Diisobutyl adipate	24.3	340	300	810	< LOD	98	< LOD	310	280	220	< LOD	450	< LOD	

* All compounds were tentatively identified, except the terpenes that were identified using authentic standards as well.

Table S2. Elemental concentrations and emission factors measured in TSP using the reference method and in $PM_{2,5}$, PM_1, $PM_{0,5}$, $PM_{0,2}$ fractions obtained by the DGI impactor for the mixing stack.

		DGI					TSP reference method
		Total DGI	$PM_{2,5}$	PM_1	$PM_{0,5}$	$PM_{0,2}$	Particulate fraction
Al	Concentration (μg m^{-3})	11.79	10.99	8.34	4.70	3.95	26.15
	Blank (μg m^{-3})						12.20
	QL (μg m^{-3})	0.63	0.51	0.38	0.25	0.13	2.63
	Emission factor (g ton^{-1})	0.5	0.4	0.3	0.2	0.1	1.0
Ca	Concentration (μg m^{-3})	11.80	11.03	8.65	5.09	4.22	24.55
	Blank (μg m^{-3})						3.87
	QL (μg m^{-3})	0.32	0.25	0.19	0.13	0.06	2.40
	Emission factor (g ton^{-1})	0.5	0.4	0.3	0.2	0.2	0.9
K	Concentration (μg m^{-3})	1.28	1.18	1.04	0.77	0.65	1.25
	Blank (μg m^{-3})						0.20
	QL (μg m^{-3})	0.32	0.25	0.19	0.13	0.06	
	Emission factor (g ton^{-1})	0.04	0.04	0.04	0.04	0.04	0.05
Mg	Concentration (μg m^{-3})	36.60	32.22	27.66	19.39	17.59	27.67
	Blank (μg m^{-3})						1.97
	QL (μg m^{-3})	0.00	0.51	0.38	0.25	0.13	2.63
	Emission factor (g ton^{-1})	1.4	1.2	1.0	0.7	0.7	1.0
Na	Concentration (μg m^{-3})	3.17	2.53	1.90	1.27	0.63	4.20
	Blank (μg m^{-3})						1.59
	QL (μg m^{-3})	0.00	5.07	3.80	2.53	1.27	26.35
	Emission factor (g ton^{-1})	0.1	0.1	0.1	0.04	0.04	0.2
Si	Concentration (μg m^{-3})	62.55	57.49	47.30	30.17	19.06	-
	Blank (μg m^{-3})						-
	QL (μg m^{-3})	0.00	2.53	1.90	1.27	0.63	-
	Emission factor (g ton^{-1})	2.3	2.1	1.8	1.1	0.7	-
Ti	Concentration (μg m^{-3})	21.31	20.61	1.,73	4.43	2.24	28.70
	Blank (μg m^{-3})						0.16
	QL (μg m^{-3})	0.00	0.51	0.38	0.25	0.13	5.87
	Emission factor (g ton^{-1})	0.8	0.8	0.6	0.2	0.1	1.1
